# A comparison between observed and DFT calculations on structure of 5-(4-chlorophenyl)-2-amino-1,3,4-thiadiazole

**DOI:** 10.1038/s41598-019-55793-5

**Published:** 2019-12-17

**Authors:** Nagaraju Kerru, Lalitha Gummidi, Sandeep V. H. S. Bhaskaruni, Surya Narayana Maddila, Parvesh Singh, Sreekantha B. Jonnalagadda

**Affiliations:** School of Chemistry & Physics, University of KwaZulu-Natal, Westville Campus, Chiltern Hills, P/Bag X54001, Durban, 4000 South Africa

**Keywords:** Chemical synthesis, Synthetic chemistry methodology

## Abstract

The crystal and molecular structure of 5-(4-chlorophenyl)-2-amino-1,3,4-thiadiazole **3** was reported, which was characterized by various spectroscopic techniques (FT-IR, NMR and HRMS) and single-crystal X-ray diffraction. The crystal structure **3** (C_8_H_6_ClN_3_S) crystallized in the orthorhombic space group *Pna2*_1_ and the unit cell consisted of 8 asymmetric molecules. The unit cell parameters were *a* = 11.2027(2) Å, *b* = 7.6705(2) Å, *c* = 21.2166(6) Å, *α* = *β* = *γ* = 90°, V = 1823.15(8) Å^3^, *Z* = 8. In addition, the structural geometry (bond lengths, bond angles, and torsion angles), the electronic properties of mono and dimeric forms of compound **3** were calculated by using the density functional theory (DFT) method at B3LYP level 6-31+ G(d,p), 6-31++ G(d,p) and 6-311+ G(d,p) basis sets in ground state. A good correlation was found (R^2^ = 0.998) between the observed and theoretical vibrational frequencies. Frontier molecular orbitals (HOMO and LUMO) and Molecular Electrostatic Potential map of the compound was produced by using the optimized structures. The NBO analysis was suggested that the molecular system contains N-H…N hydrogen bonding, strong conjugative interactions and the molecule become more polarized owing to the movement of π-electron cloud from donor to acceptor. The calculated structural and geometrical results were in good rational agreement with the experimental X-ray crystal structure data of 1,3,4-thiadiazol-2-amine, **3**. The compound **3** exhibited n→π* UV absorption peak of UV cutoff edge, and great magnitude of the first-order hyperpolarizability was observed. The obtained results suggest that compound **3** could have potential application as NLO material. Therefore, this study provides valuable insight experimentally and theoretically, for designing new chemical entities to meet the demands of specific applications.

## Introduction

Computational chemistry is playing an increasingly important role in the fields of chemical, biological and material sciences^1^. In organic chemistry, it especially helps in understanding the molecular structure, giving an insight into the reaction pathways and chemical mechanisms through estimation of the geometrical properties of compounds^2,3^. This can provide detailed electronic properties of reactants, intermediates and products, and could also help the comparison with their diverse experimental studies^4–6^. The growing associations between experimental and computational chemistries have solved an extensive range of organic problems^7^. Recently, the Dentistry Functional Theory (DFT) has made considerable advances in organic synthesis and is used for computing the electronic and geometrical properties^8,9^. The computational chemistry could kindle the new ideas in planning novel chemical reactions and mechanistic studies.

*N*-heterocyclic compounds with five-membered rings are the most frequently encountered building blocks in both organic and medicinal chemistry, possessing a number of biological activities^10^. Amongst the five-membered heterocyclic compounds, the 1,3,4-thiadiazole derivatives has attracted scientific community, due to their unique structural features^11^. Over the years, these 1,3,4-thiadiazole derivatives have drawn much attention in the fields of medicinal chemistry^12,13^, material chemistry^14^ and agriculture^15,16^, as different molecules have exhibited varied biological activities, such as antioxidant and antiproliferative^17^, anti-tubercular^18^, antiviral^19^, anticancer^20^, and antibacterial activities^21^. In addition, these thiadiazole compounds have also been a valuable source as reaction intermediates in the synthesis of commercially available drugs (megazol and furidiazine)^22^.

In view of the broad ranging applications of 2-amino-1,3,4-thiadiazole scaffold and in extension to our efforts to develop various biologically active conjugates^23^, we conducted the comparative studies between experimental and theoretical (DFT) data of 5-(4-chlorophenyl)-2-amino-1,3,4-thiadiazole, a molecule with anti-proliferative activity, which was earlier reported by Guan *et al*.^24^. The crystal structure and theoretical calculations of this compound have not been reported yet. Here, we have synthesized the 5-(4-chlorophenyl)-1,3,4-thiadiazol-2-amine (**3**) according to the reported literature, and characterized by using crystal X-ray, IR, NMR, HRMS. In addition, the optimized geometry parameters of the title compound was carried out at DFT and TD-DFT methods with the B3LYP functional and two basis sets 6-31+ G(d,p) and 6-311+ G(d,p), and conceivable correlations were investigated between observed and theoretical data.

## Results and Discussions

### X-ray crystal structure description and synthesis of compound 3

**Figure 1:**
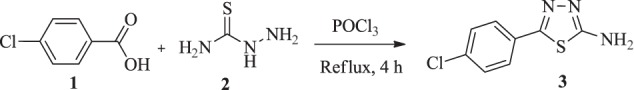
Synthetic path way for 1,3,4-thiadiazol-2-amine (**3**).

**Figure 2:**
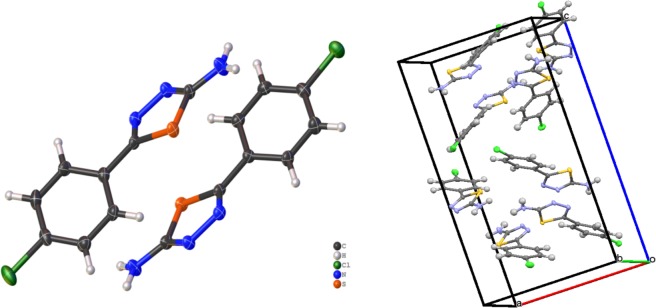
X-ray molecular structure of compound **3**, ORTEP view at the 50% probability level of thermal ellipsoids, packing diagram, when viewed down the *b*-axis.

**Figure 3:**
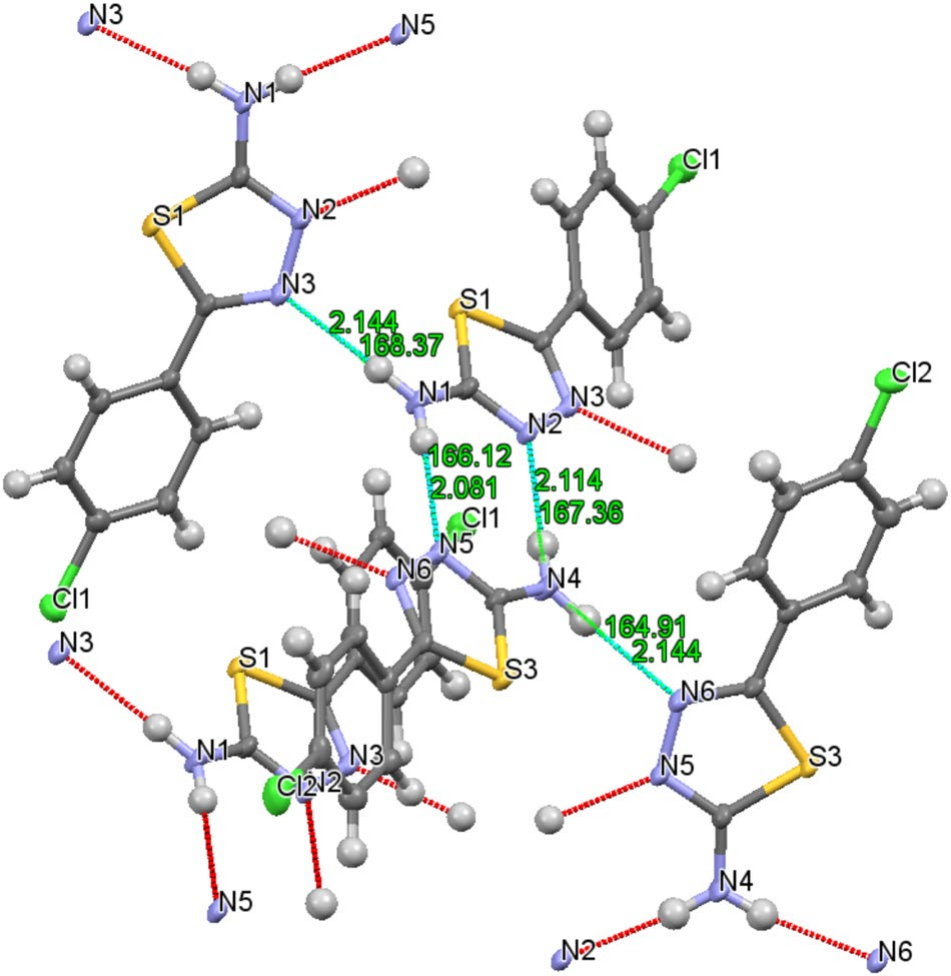
Dotted lines showing intermolecular hydrogen bondings (N-H…N).

**Figure 4:**
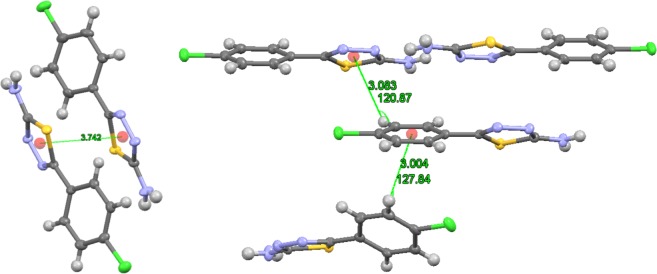
Molecular stacking representation emphasizing π/π and C-H…π bond interaction between molecules.

Figure 1 depicts the synthesis of 5-(4-chlorophenyl)-1,3,4-thiadiazolo-2-amine (**3**) compound. It was achieved by the reaction between the corresponding 4-chlorobenzoic acid (**1**) and thiosemicarbazide (**2**). In this process, using phosphorous oxychloride as an acid activating agent, the reaction mixture was refluxed for 4 h as per reported procedure^25^. The structural assignment of the purified compound (**3**) was established and confirmed by employing various spectroscopic techniques. The identified title molecule (**3**) was subjected to single-crystal X-ray diffraction analysis. The comprehensive X-ray crystallographic evidences were placed in Cambridge crystallographic data center (CCDC 1879792). A single colorless plank-shaped crystal of compound,**3** was recrystallized through sluggish evaporation of hot ethanol over a period of five days. For the crystallographic analysis, Fig. 2 represented the 50% probability of ORTEP image and the structural established crystal data are illustrated in Table 1. The synthesized title molecule (**3**) develops in the orthorhombic space group *Pna2*_1_ by *Z* = 8, and Table 2 represented the measured chosen torsion angles, bond distances and angles. The crystal compound embraces of two independent similar molecules in the asymetric unit and the heteroaromatic rings were planar. The dimers with 4-chlorophenyl and 1,3,4-thiadiazol-2-amine rings were bonded through C2-C3 and C10-C11 junction with a bond length of 1.464(5) and 1.469(5) Å. These were approximately equal bond lengths, which were within estimated range for C-C (1.467(4) Å)^26,27^ (Fig. 2). The bond angles of these rings exhibited 121.0(3)° for C4-C3-C2 and 120.6(3)° for C12-C11-C10. Moreover, the C=N double bonds were conjugated with the unshared electron pairs on the N1 and S1 atoms of compound **3**, and the corresponding endocyclic (N=C) bond lengths were almost similar with N2-C1 (1.316(4)), N3-C2 (1.296(4)), N5-C9 (1.324(4)) and N6-C10 bonds (1.303(4)) Å. The carbon and carbon bonds (C-C) in the 4-chlorophenyl ring were of almost equal lengths in the range of 1.371 to 1.399 Å. In addition, similar conformations of four sets of asymmetric units were identified in the unit cell along the *b* axis of crystal packing diagram, which were linked over intermolecular symmetric hydrogen bond interactions (N-H…N) (Figs. 2 and 3). The strongest intermolecular hydrogen bonding existed between the protons of the exocyclic amino moiety and the nitrogen atoms of the 1,3,4-thiadiazole ring with the bond distance of 2.11 Å N(4)-H(4 A)…N(2) (167.3°) and 2.08 Å N(1)-H(1 A)…N(5) (166.1°), and other amine hydrogen atom extended the supramolecular network (N(1)-H(1B)…N(3) and N(4)-H(4B)…N(6)) with other nitrogen atoms of the thiadiazole ring (Table 3). Further, the π-π stacking of the crystal structure **3** was found with 3.742 Å space among the centroids of the thiadiazole rings. The aromatic C-H…π interaction with distance (H…centroids) of 3.083 Å and 3.004 Å, and the angles were (C-H…centroids) 120.8° and 127.8°, respectively (Fig. 4).

**Table 1:** Crystal data and structure refinement for compound **3**.

Formula	C_8_H_6_ClN_3_S
Formula Weight	211.67
*D*_*calc*._/g cm^−3^	1.542
*μ*/mm^−1^	0.599
Shape	plank
Colour	colourless
Crystal System	orthorhombic
Size/mm^3^	0.27 × 0.17 × 0.09
*T*/K	100(2)
Space Group	*Pna*2_1_
Flack Parameter	−0.07(5)
Hooft Parameter	0.17(12)
*a*/Å	11.2027(2)
*b*/Å	7.6705(2)
*c*/Å	21.2166(6)
*α*/^°^	90
*β*/^°^	90
*γ*/^°^	90
*Z*	8
*Z*′	2
*V*/Å^3^	1823.15(8)
Radiation type	MoK_*α*_
Wavelength/Å	0.71073
F(000)	864.0
Crystal size/mm^3^	0.27 × 0.17 × 0.09
*Θ*_*min*_/^°^	3.219
*Θ*_*max*_/^°^	28.415
Index ranges	−14 ≤ h ≤ 14, −10 ≤ k ≤ 9, −27 ≤ l ≤ 16
Independent Refl.	3123
Measured Refl.	7100
Reflections with I > 2(I)	2968
*R*_*int*_	0.0279
Restraints	1
Parameters	236
Largest Peak	1.030
GooF^2^	1.081
Deepest Hole	−0.261
*wR*_2_ (all data)	0.1037
*wR*_2_	0.1020
*R*_1_ (all data)	0.0423
*R*_1_	0.0401

**Table 2:** Selected structural parameters.

Atom	Experimental	Calculated B3LYP/6-31+ G(d,p)		Calculated B3LYP/6-311+ G(d,p)	
		Monomer	Dimer	Monomer	Dimer
***Bond length (Å)***					
S1-C1	1.738(4)	1.758	1.769	1.757	1.763
S1-C2	1.743(3)	1.779	1.782	1.779	1.779
S3-C9	1.741(4)	—	1.755	—	1.757
S3-C10	1.744(3)	—	1.779	—	1.772
Cl1-C6	1.725(4)	1.756	1.757	1.756	1.758
Cl2-C14	1.739(4)	—	1.754	—	1.753
N1-C1	1.340(4)	1.371	1.357	1.371	1.355
N2-N3	1.381(4)	1.363	1.362	1.361	1.359
N4-C9	1.333(5)	—	1.363	—	1.367
N5-N6	1.377(4)	—	1.365	—	1.364
C2-C3	1.464(5)	1.466	1.466	1.465	1.465
C10-C11	1.469(5)	—	1.466	—	1.465
N2-C1	1.316(4)	1.308	1.313	1.303	1.309
N5-C9	1.324(4)	—	1.313	—	1.303
N6-C10	1.303(4)	—	1.300	—	1.299
C2-N3	1.296(4)	1.302	1.300	1.296	1.296
***Bond angles (***^***o***^***)***					
C1-S1-C2	86.66(16)	86.0	86.1	85.9	86.0
C9-S3-C10	87.27(16)	—	86.3	—	86.2
C1-N2-N3	111.8(3)	112.6	112.8	112.7	112.8
C9-N5-N6	112.2(3)	—	113.0	—	112.6
N1-C1-S1	114.2(3)	122.5	121.6	122.4	122.1
N2-C1-N1	124.6(2)	123.2	124.7	123.3	124.1
N3-C2-C3	124.6(3)	124.1	124.2	124.2	124.1
N4-C9-S3	122.6(3)	—	122.5	—	122.4
N5-C9-N4	124.0(3)	—	123.7	—	123.5
N6-C10-C11	124.1(3)	—	124.3	—	124.8
C4-C3-C2	121.0(3)	119.2	119.3	119.2	122.1
C12-C11-C10	120.6(3)	—	119.3	—	121.5
C5-C6-Cl1	120.4(3)	119.5	119.5	119.5	119.5
C13-C14-Cl2	119.7(3)	—	119.5	—	119.4
***Torsion angles (***^***o***^***)***					
C1-S1-C2-C3	180.3(3)	−179.1	−178.8	−179.2	−179.3
C1-N2-N3-C2	1.3(5)	0.5	0.5	0.5	0.4
C2-S1-C1-N1	178.4(94)	−176.4	−177.9	−176.4	−177.1
C3-C2-N3-N2	179.4(3)	178.9	178.7	178.9	179.1
C9-N5-N6-C10	0.6(5)	—	−0.4	—	−1.0
C10-S3-C9-N4	179.5(4)	—	176.7	—	176.1
C4-C5-C6-Cl1	−179.6(3)	179.9	179.8	179.9	179.9
C12-C13-C14-Cl2	−179.5(3)	—	−179.9	—	−179.9

**Table 3:** Intermolecular hydrogen bonding geometry of the compound **3** (Å, °).

D-H…A	d(D-H)/Å	d(H…A)/Å	d(D…A)/Å	∠D-H…A/°
**Experimental**				
N(1)-H(1 A)…N(5)^a^	0.88	2.08	2.943(4)	166.1
N(4)-H(4 A)…N(2)^b^	0.88	2.11	2.979(4)	167.3
**Calculated B3LYP/**6-**31** **G(d,p)**				
N(1)-H(1 A)…N(5)	1.009	2.254	3.031	162.6
N(4)-H(4 A)…N(2)	1.012	2.266	3.016	167.7

Symmetry codes: ^a^ + X,−1 + Y, +Z; ^b^ + X,1 + Y, +Z.

Further, in the IR spectrum (Table 4), 1,3,4-thiadiazole (**3**) displayed prominent absorption peak at 3255 cm^−1^ corresponds to NH_2_ group of 1,3,4-thiadiazol-2-amine ring, and its ^1^H NMR spectrum (Table S1) resonance signals showed a singlet at *δ* 7.46 ppm, corresponds to the two protons of NH_2_ group. While the two set of doublets appeared at *δ* 7.77 (*J* = 8.6 Hz) and 7.52 (*J* = 8.6 Hz) ppm correspond to the four protons of 4-chlorophenyl ring. The most prominent peak appeared at *δ* 168.8 and 155.12 ppm in ^13^C NMR spectrum likely belongs to the 1,3,4-thiadiazole ring carbons. The molecular-ion peak finding at *m/z* = 212.0585 in the HRMS spectrum.

**Table 4:** Most characteristic experimental and calculated vibrational frequencies (cm^−1^) and assignments of the compound **3** at B3LYP/6-31+G(d,p).

Assignments	Experiment (cm^−1^)	Calculated (cm^−1^)	
		Monomer	Dimer
*υ*NH_2_	3255	3228	3219
*υ* = CH_aromatic_	3082	3189	3188
*υ*C=N	1629	1641	1652
*υ*C=C_benzene ring_	1597	1610	1611
*υ* = C-N	1261	1281	1283
*υ*C-S-C	735	732	733
*υ*C-Cl	706	725	724

### DFT optimization investigation

**Figure 5:**
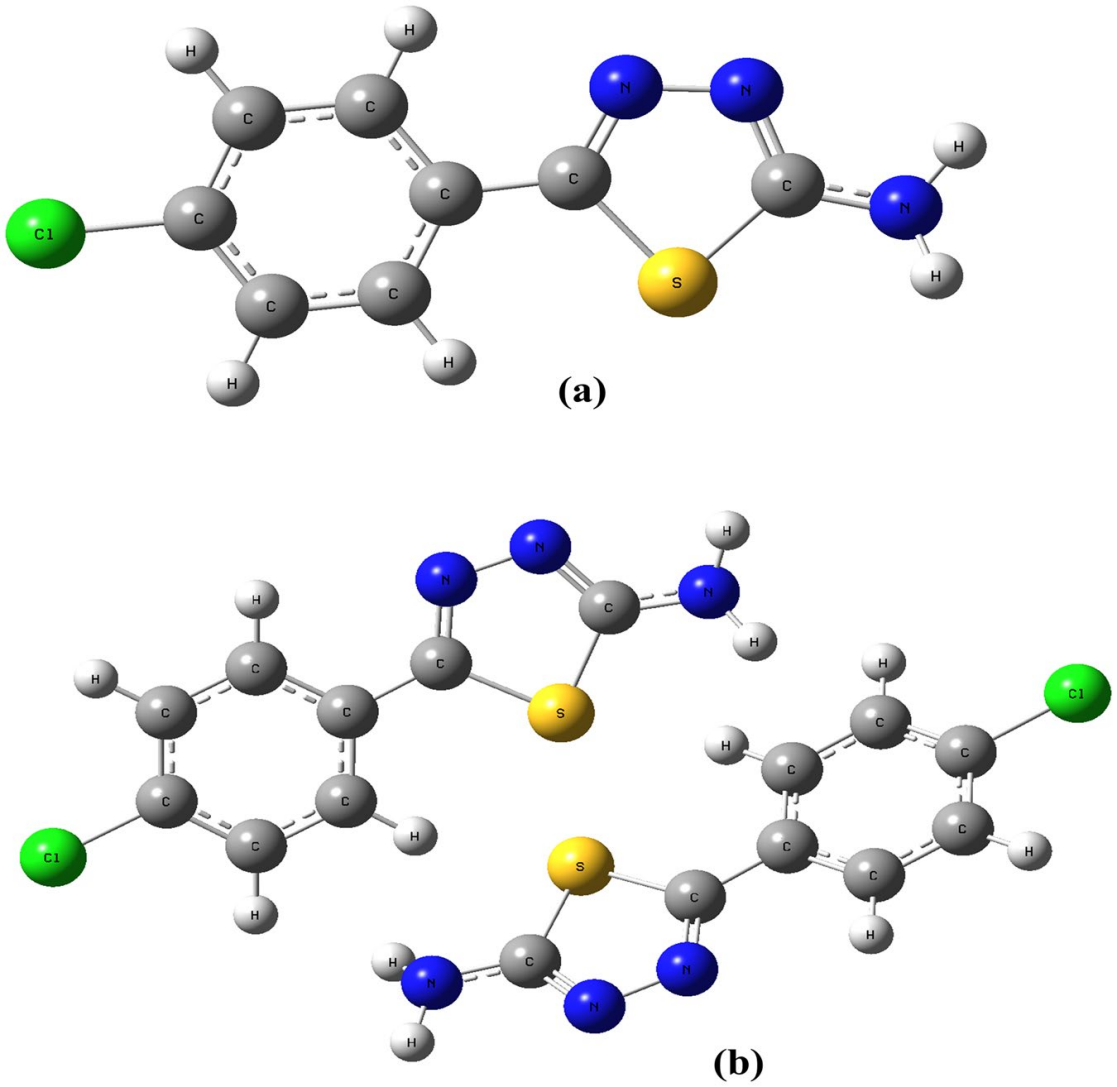
Optimized structure of monomer (a) and dimer (b) of compound **3** obtained by B3LYP/6-31+ G(d,p).

The most appropriate density functional theory based function is B3LYP with 6-31+ G(d,p) and 6-311+ G(d,p) basis sets were used for creating the optimized geometries of monomer and dimeric form of molecule **3** in the gas phase (Fig. 5). The computed geometrical parameters (torsion angles bond lengths and angles) are represented in Table 2 and these parameters were compared with the found experimental parameters. The identified bond lengths were good reliable with the experimental parameters through an average divergence of 0.01 Å to 0.03 Å for both basis sets. The deviations could take place by the intramolecular hydrogen bonding interactions. It was concluded that the sequences of B3LYP functional with both 6-31+ G(d,p) and 6-311+ G(d,p) basis sets produce the best compatible results with the experimental values (Table 3).

The calculation of the vibrational frequencies in IR-spectrum of monomer and dimeric forms of molecule **3** was carried out by using B3LYP function with 6-31+ G(d,p) basis set. The chosen characteristic vibrational band values and along with observed values are presented in Table 4. In IR spectrum, the N-H stretching frequency was appeared at 3255 cm^−1^, this same band was identified at 3219 cm^−1^ and 3228 cm^−1^ for dimeric and monomeric of compound **3**. The computed vibrational value was in excellent agreement with the observed value. In addition, the benzene ring of C-H stretching IR band was seemed at 3082 cm^−1^ (calculated as monomer 3189 cm^−1^ and dimer 3188 cm^−1^). The most prominent vibrational stretching absorbance was found at 1629 cm^−1^ due to the endocyclic group C=N group of the thiadiazole ring and the computed as 1641 cm^−1^ and 1652 cm^−1^ for monomer and dimer forms. The strong stretching band observed for C=N group, this may be due to the neighbouring atoms of electronic effects (unshared electron pairs on the S, N atoms) and intermolecular hydrogen bonding^28^. While the small deviation of computed C=C vibration band (1610 cm^−1^ for monomer and 161 cm^−1^ for dimer) was identified, as compared with the observed value (1597 cm^−1^).

**Figure 6:**
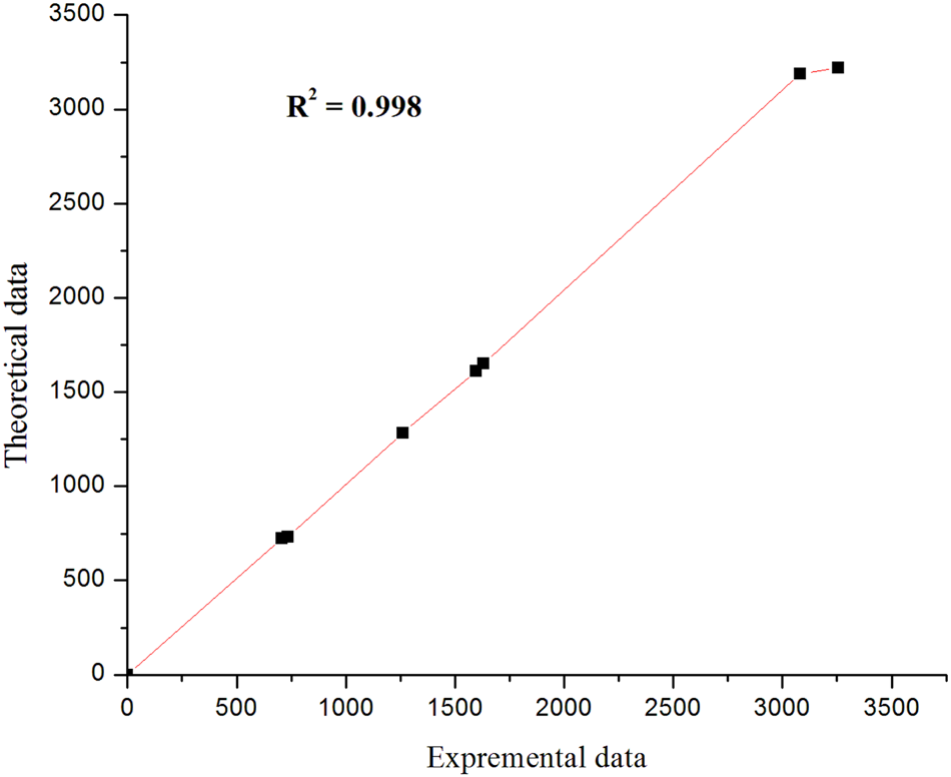
Correlation studies between observed and calculated IR vibrational frequencies of compound **3**.

The C-N vibrational stretching calculated at 1281 cm^−1^ for monomer and 1283 cm^−1^ for dimeric form and the band was observed at 1261 cm^−1^. The C-S-C stretching obtained at 732 cm^−1^ and 733 cm^−1^ for the mono and dimeric forms of the 1,3,4-thiadiazole moiety and which were almost matched with observed vibrational mode at 735 cm^−1^. The computed C-Cl stretching absorbance band (725 cm^−1^ for monomer and 724 cm^−1^ for dimer) was in good agreement with the observed value (706 cm^−1^). The correlation studies were summarized in Fig. 6 and these results indicates the good correlation founded (R^2^ = 0.998) amongst the experimental and calculated vibrational absorbance values. The acquired vibrational frequency values were consistence with results obtained in the literature^29,30^.

The calculated ^1^H and ^13^C NMR spectrum of the structure **3** was analyzed by using B3LYP/6-31+ G(d,p) in DMSO and the chemical shift (^1^H and ^13^C NMR) values are presented in Table S1. A good agreement between the observed and calculated chemical shift values, except for nitrogen atom bonding with H1A and H1B protons. The calculated deviation was observed due to the hydrogen bonding interactions (N-H…N) and was given in Table 3.

### Molecular electrostatic potential (MEP) investigation

The MEP considered as an interaction energy at a certain zone of a structure was involved in the electrical charge distribution from the proton, nuclei and electrons, which were positioned at *r*.

The *V(r)* was obtained from the following the Eq. (1)^31^.

1 $$V(r)={\sum }_{A}\frac{{Z}_{A}}{({R}_{A}-r)}-\int \frac{\rho (r^{\prime} )}{|r^{\prime} -r|}dr^{\prime} $$

Where *Z*_*A*_ signifies the charge of the nucleus A, which is positioned at *R*_*A*_, (*r*′) denote the dummy integration variable and *ρ*(*r*′) represent the electron density function of the structure.

**Figure 7:**
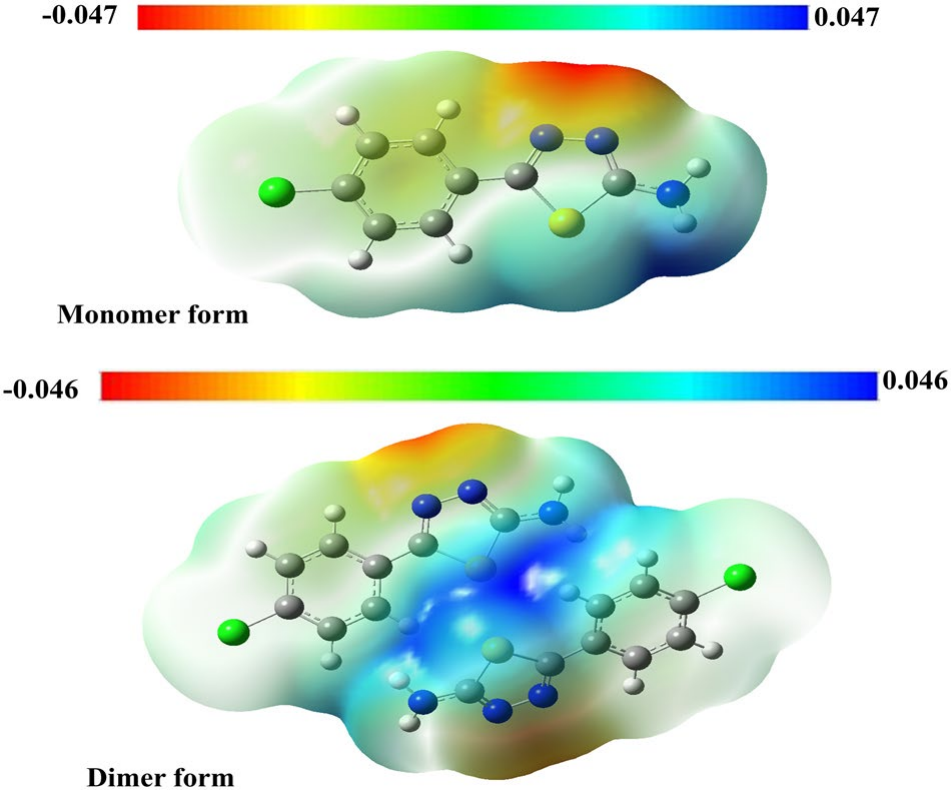
Molecular electrostatic potential (MEP) analysis of compound **3**.

The probable nucleophilic (blue region) and electrophilic (red region) attack sites were established. The MEP was computed at the B3LYP functional and 6-31+ G(d,p) level of theory of monomeric and dimeric forms, and the images displayed in Fig. 7, which gives the valuable evidence about the intramolecular hydrogen bonding interactions and the possible nucleophilic and electrophilic sites^32^. The higher negative region was placed on the N-N atoms of the thiadiazole ring (red, Fig. 7), which was regarded as the feasible position for electrophilic attack with *V(r)*of −0.046 a.u, due to the N-H–N hydrogen bonding interfaces around this zone. The supreme positive zone was positioned at the amino group of nitrogen and sulfur atoms of thiadiazole ring of the molecule with *V(r)* of 0.046 (Fig. 7). This shows the probable site for nucleophilic attack on unshared electron pair atoms (N and S).

### Frontier molecular orbital (FMO) analysis

**Figure 8:**
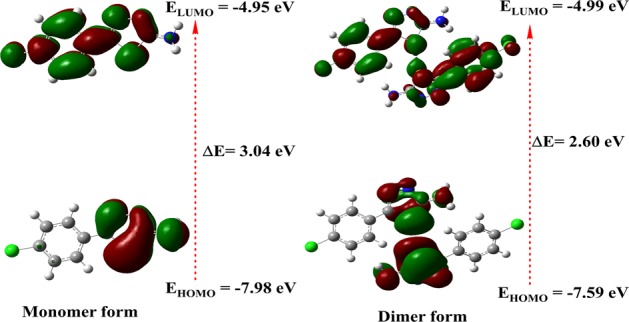
HOMO–LUMO energy diagram of compound **3**.

The electric and optical parameters of the structure in the FMO theory was computed by using B3LYP/6-31+G(d,p) and B3LYP/6-311+G(d,p) basis sets. FM orbitals are the important orbitals in a structure, and it is considered as the lowest molecular orbitals (LUMO) and the highest molecular orbital (HOMO)^29,33^. The outermost orbital occupied by electrons called the HOMO, which can act as an electron donors. The energy of the LUMO is the foremost vacant innermost orbital unoccupied with electrons, it performs as an electron acceptor. The electrons donating capacity of molecule is allied to the *E*_HOMO_, and the over-all the greater the HOMO energy (smaller negative value), the superior the capacity to donate electrons^29,34^. The energy difference (ΔE) between the HOMO and LUMO, softness, electronegativity, chemical hardness and electrophilicity index values of monomer and dimer of compound **3** are presented in Table 5 and Fig. 8. The HOMO orbital was displayed charge density localized around the 2-amino-1,3,4-thiadiazole ring, while a charge density localized on the 4-chlorophenyl ring in the LUMO orbitals of the both monomer and dimeric forms of both basis sets, signifying that these moieties can incentive the electron transition. The energy gap between the HOMO and LUMO orbitals indicates the chemical strength and reactivity of the molecule^35,36^. The energy gap difference between the HOMO and LUMO is ΔE of 3.03 and 3.01 eV for monomer of and ΔE of 2.59 and 2.93 eV for dimer of B3LYP/6-31+ G(d,p) and B3LYP/6-311+ G(d,p) basis sets. The energy of the smaller band space gap increases the reactive nature and stability of compound **3**. In addition, the compound characterized based on reactivity, chemical hardness (*η*) electronegativity (*μ*), softness (*ζ*) and electrophilicity index (*Ψ*) were determined according to Eqs. (2–5).

2 $${\rm{Chemical}}\,{\rm{hardness}}\,\eta =\frac{{\rm{I}}-A}{2}$$

3 $${\rm{Softness}}\,{\rm{\zeta }}=\frac{1}{2{\rm{\eta }}}$$

4 $${\rm{Electronegativity}}\,\mu \approx -=-\,\frac{{\rm{I}}+A}{2}$$

5 $${\rm{Electrophilicity}}\,{\rm{index}}\,{\Psi }=\frac{{{\rm{\mu }}}^{2}}{2{\rm{\eta }}}$$

Where A and I is electron affinity and ionization potential.

$${\rm{A}}=-\,{{\rm{E}}}_{{\rm{LUMO}}}\,{\rm{and}}\,{\rm{I}}=-\,{{\rm{E}}}_{{\rm{HOMO}}}$$

**Table 5:** The calculated energies of the compound **3**.

Quantum descriptors	B3LYP/6-31+ G(d,p)		B3LYP/6-311+ G(d,p)	
	Monomer	Dimer	Monomer	Dimer
E_LUMO_ (eV)	−4.95	−4.99	−5.02	−5.02
E_HOMO_ (eV)	−7.98	−7.59	−8.03	−7.96
ΔE (eV)	3.04	2.60	3.01	2.93
μ (eV)	6.46	6.29	6.53	6.49
η (eV)	1.52	1.30	1.50	1.46
ζ (eV)	0.32	0.38	0.33	0.34
Ψ (eV)	13.72	15.21	14.15	14.33

As can be seen in the Table 5, the title compound can be referred as a soft molecule, since lesser energy gap systems are termed as soft molecules. The chemical behavior of the compound was identified by electronegativity factor. The greater value of the electronegativity of molecule **3** is a designated of its chemical reactivity.

### Electronic absorption spectrum analysis

**Figure 9:**
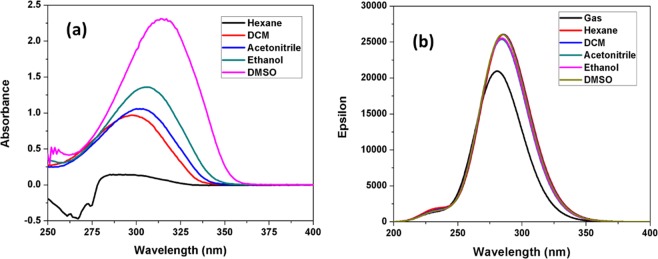
(**a**) Experimental absorption spectrum of the titled compound (**3**) in different solvents at room temperature; (**b**) TD-DFT calculated absorption spectrum in the gas phase and different solvents.

The electronic spectral analysis of the title structure was computed in both gas and various organic solvent phases by using time dependent-DFT (TD-DFT) IEFPCM model at the B3LYP/6-31++G(d,p) level theory on the ground state optimized geometry. Although, the observed absorption spectra were investigated at room temperature in the diverse solvents with 5×10^−5^ M of a concentration, and the plots are presented in the Figs. 9a,b. The experimental maximum wavelength (*λ*_*max*_) and absorbance was measured at room temperature and the values are summarized in Table 6. Dipole moment (*μ*, D), major absorption energy *E*, oscillator strength (*f*), maximum wavelength (*λ*_*max*_), electronic transition of excitation energy and atomic orbital contribution were calculated in solvent and gas phases, and the values are demonstrated in Table 7.

**Table 6:** Experimental electronic absorption band of the titled compound (**3**) in different solvents at room temperature.

S.No	Solvent	Wavelength λ_max_ (nm)	Absorbance
1	Hexane	290	0.1438
2	DCM	297	0.9681
3	Acetonitrile	300	1.0589
4	Ethanol	307	1.3583
5	DMSO	314	2.3132

**Table 7:** Calculated energies, dipole moments (D), maximum absorption wavelengths (*λ*_*max*_), excitation energies (eV), oscillator strengths (*f*), assignment of electronic transitions (HOMO(H)→LUMO(L)) and major contribution (%) for the titled compound **3** in gas phase and in different solvents.

Solvent	Parameters						
	E_Total_ (a.u)	Dipole moment	*λ*_*max*_	*f*	Transition energy	Electronic transition	Major % contribution
Gas phase	−1331.0686	2.43	280.97	0.5110	4.4127	H→ L	96.71
			272.48	0.0004	4.5502	H-1→ L	95.50
Hexane	−1331.0762	2.84	285.88	0.6395	4.3369	H→ L	97.91
			269.05	0.0005	4.6083	H-1→ L	94.89
DCM	−1331.0818	3.43	285.37	0.6405	4.3447	H→ L	97.95
			264.87	0.0005	4.6810	H-2→ L	94.01
Acetonitrile	−1331.0830	3.63	284.44	0.6232	4.3588	H→ L	97.86
			263.76	0.0062	4.7006	H-1→ L	56.36
Ethanol	−1331.0828	3.60	284.64	0.6271	4.3559	H→ L	97.88
			263.83	0.0006	4.6993	H-2→ L	92.50
DMSO	−1331.0835	3.65	285.12	0.6398	4.3485	H→ L	97.96
			263.80	0.0063	4.6999	H-1→ L	56.70

The observed absorption spectrum of the title compound was recorded in various solvents such as hexane, dichloromethane (DCM), acetonitrile, ethanol and DMSO, concluded that the very low solubility was observed in hexane solvent. As results from Fig. 9a, the molecule was showed high intensity band at 290, 297, 300, 307 and 314 nm in all the five solvents, which can be attributed to the n→π* electronic transition. This was occurred from the molecular orbitals of the auxochrome (NH_2_) of exocyclic nitrogen lone pairs and the conjugated π-bond of azomethine (-C=N-N=C-) of 1,3,4-thiadiazole moiety. With the increasing the solvent polarity from non-polar to polar, n→π* transition get slightly shifted towards longer wavelength, which was probably due to the solvatochromic effect (Tables 6 and 7)^37^. Compound **3** displayed a positive absorption solvatochromism, which signifies that the absorption was redshifted with cumulative solvent polarity. The highest energy transition was noticed in polar solvents such as DMSO and ethanol, resulting in enhancement of dipole moment (for ethanol 3.60 D and DMSO 3.65 D).

Consequently, the calculation was further engaged with TD-DFT-IEP-PCM model^38^. A strong absorption band was observed at 280 nm (*f* = 0.5110) and 285 nm (*f* = 0.6398) in the gas and solvent phase (DMSO), which corresponds to the excitation of electrons occurred from HOMO to LUMO transition (96.71% and 97.96% orbital contribution). It could be owing to the solvent effects, which can impact the geometry, molecular properties and electrostatic structure, thus causing shift in absorption bands^39^. The electronic transitions occurred from dipolar interaction with solvents of different polarities suggest that the intramolecular charge transfer (ICT) of the emission state in which the HOMO and LUMO were apparently localized on the 1,3,4-thidiazole moiety and the phenyl ring.

**Figure 10:**
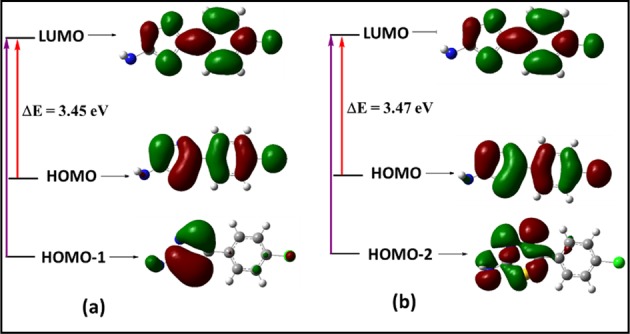
Frontier molecular orbitals contributing in electronic absorption along with band gap (ΔE) in the solvent phase (**a**) DMSO and (**b**) DCM for the titled compound **3**.

The distribution of energy levels and electronic transitions of molecular orbitals in the title compound in both solution and gas phases are overlapping. The frontier MO analysis in calculating the energies and the nature of frontier MOs indicate that these orbitals are the maximum feasible sites of interchange density, directing to the interaction between orbitals. There was a small variance in the energy gap in gas and solvent phases (Fig. S1). The estimated energy gap (ΔE = HOMO-LUMO) value in solvent phase for DMSO and DCM were 3.45 and 3.47 eV respectively, which corresponds to the intramolecular charge transfer (S_0_→S_1_) transition (Fig. 10a). It may be assumed that for DCM and DMSO could stabilize the compound, due their different polarities and dielectric constants by elevating the energy gap between their HOMO and LUMOs. The energy gap decreased with increasing solvent polarity, which shifted the absorption peak to longer wavelength than 290–314 nm, consequently leading to decline in their absorption capacity in the desired UV range. The HOMO orbital shows that π-bonding and non-bonding (n-type) nature orbitals were identified on the thiadiazole moiety, phenyl ring and nitrogen atom of exocyclic amino group, the HOMO-1 orbital was localized on the 2-amino-1,3,4-thiadiazole moiety and chlorine atom, and the HOMO-2 orbital was localized on the 2-amino-1,3,4-thiadiazole moiety only. Similarly, the LUMO shown the π* anti-bonding type orbitals on carbon, sulfur and nitrogen atoms were localized in the phenyl and thiadiazole ring moieties. Therefore, the results concluded that the solvent polarity enhances the probability of transition (n→π*) in the present compound. A very good correlation was observed between the TD-DFT and experimental absorption spectra results.

### Mulliken atomic charges

**Figure 11:**
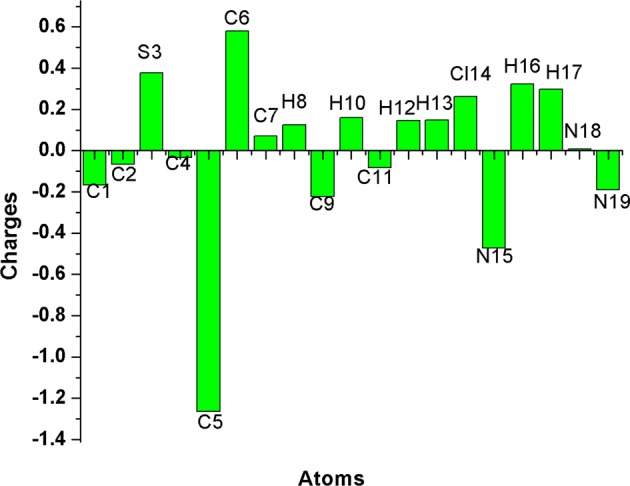
Bar diagram representing the charge distribution of compound **3**.

Mulliken atomic charge results takes an imperative position in the applications of quantum chemical calculation, as the molecular polarizability, atomic charges affect the dipole moment and electronic structural features of molecular systems. The development of donor and acceptor pairs of charge transfer was succeeded, because of charge distributions over the atoms in the present molecule. Mulliken population investigation of monomer and dimeric forms of compound **3** was estimated by using function B3LYP with 6-31+ G(d,p) basis set and results are demonstrated in Tables S2 and S3 (**ESI**). Figure 11 illustrates the charge distribution structure of compound **3**. The charge distribution presented that the all hydrogen atoms were concentrated on positively charged. The H16 (+0.322236e) and H17 (+0.298055e) atoms in particularly have higher positive atomic chargers than the other hydrogen atoms in title molecule. This was due to the presence of electron negative nitrogen (N15) atom of the exocyclic amino group. These hydrogen atoms appeal to the positive charge from the electron-withdrawing nature of the nitrogen atom. Hydrogen attached carbon atoms (C5 and C9) and quaternary carbon atoms (C1, C2, C4 and C11) exhibited negative chargers, while, the enduring carbon atoms appeared as positively charged. Another positive charge was noted on the sulfur atom, which was in the geometric center of 1,3,4-thiadiazole moiety. Nitrogen atoms (N15 and N19) were considered as more basic sites and displayed the negative atomic chargers. The existence of net positive charge on the hydrogen atom and high negative charge on the nitrogen atom, could facilitate the charge transfer over the formation of intermolecular hydrogen bonds (N-H–N). Similarly, the dimeric form of compound **3** was also calculated and same pattern of charge distribution as related to monomeric form was found (Figs. S2–4**)**.

### NBO analysis

**Figure 12:**
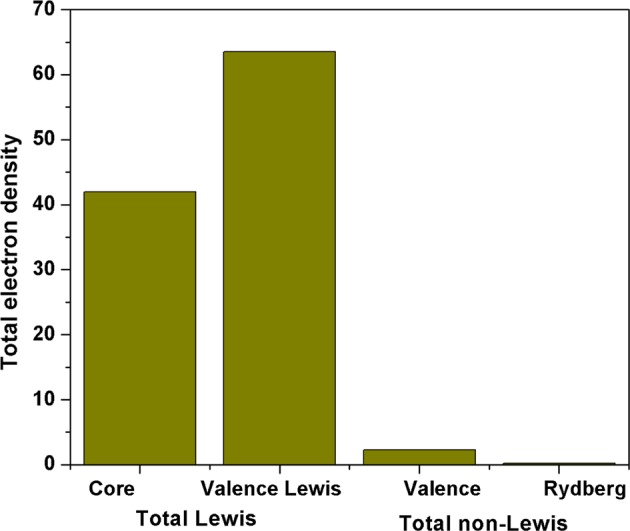
Schematic representation in terms of percentage contribution of natural population analysis and natural Lewis structure of the titled compound **3**.

Furthermore, the natural bond orbital (NBO) study of the title molecule **3** was computed at B3LYP/6-31+ G(d,p) level of model. To understanding into delocalization of electron density, intra- and inter-molecular bonding relations between the bonds, and investigate the charge transfer (CT) or conjugative relations in molecular structure^40^. The structure of the calculated compound was exhibited a type of Lewis structure (97.7%), 2.12% of valence non-Lewis, and 0.16% of Rydberg non-Lewis (Fig. 12 and Table S4). The natural Rydberg basis (NRB) populations and natural minimum basis (NMB) was also calculated in the reported compound, which was exhibited the 99.6% of maximum percentage contribution of the NMB to the molecular charge allocation.

The second-order perturbation theory study of the Fock matrix was used to assess the donor-acceptor interactions in the NBO investigation. The strength of interaction stabilization energy *E*^*(2)*^ allied with electron delocalization among the donor (*i*) and acceptor (*j*) (*i*→*j*) was assessed by the following Eq. (6).

6 $${E}^{(2)}=\Delta {E}_{ij}={q}_{i}\frac{F{(i,j)}^{2}}{{\varepsilon }_{j}-{\varepsilon }_{i}}$$

where *q*_*i*_ is the donor orbital occupancy, *F(i, j)* is the off diagonal NBO Fock matrix element, and *ε*_*j*_ and *ε*_*i*_ are implies diagonal elements (orbital energies).

The greater stabilization energy *E*^*(2)*^ value indicates more intensive interaction amongst the electron acceptors and donors, *i.e*. the high electrons donating ability and superior degree of conjugation of the entire system^39^. Numerous donor and acceptor interactions were identified for the phenyl and 1,3,4-thiadiazole rings, and lone pairs (n) of the sulfur, chlorine and nitrogen atoms. The potential intensive interactions and electron dentistry transfer from lone pair electrons to the anti-bonding orbitals along with the stabilization energy values of the compound **3** were tabulated in Table 8. These results reveal that the intramolecular interactions have occurred due to the overlap between bonding lone pair of nitrogen (LP(1)N15) and sulfur (LP(2)S3) and antibonding C2-N19 orbitals, which results in the intramolecular charge transfer (ICT) initiated stabilization of the molecular structure. Within the 1,3,4-thiadiazole fragment the n(LP(1)N15)→π*(C2-N19) and n(LP(2)S3)→π*(C2-N19) interactions facilitated strong stabilization to the molecular system of the studied compound by 156.64 kJ/mol and 114.72 kJ/mol respectively, which result in increase in electron density distribution and thus, failing of the respective bonds. This higher energy provided the stabilization to the molecular structure and these interactions suggestively impact the crystal packing with this molecule. In parallel technique calculations, the electron transfer from π(C4-C5)→π*(C7-C11) gave a stabilization energy of 88.74 kJ/mol. This interaction was owing to the π electron delocalization in a molecular system. NBO analysis further provided an evidence for intermolecular hydrogen bonding, the stabilization of energy of 3.51 kJ/mol (n→σ*) obtained from lone pair of nitrogen (LP(1)N19) to σ*(N15-H16), signifying the existence of intermolecular N-H…N hydrogen bonding. The charge delocalization occurred from the lone pair of nitrogen atom to C-S bond through n→π* interaction. The involved N18 atom lone pair 1 and N19 atom lone pair 1 to π*(C1-S3) and π*(C2-S3) interactions were provided corresponding stabilization energies of 69.07 kJ/mol and 65.35 kJ/mol, respectively, which displayed hyper conjugative interaction. Thus, the NBO study suggested that presented molecule contains strong conjugative interactions and N-H…N hydrogen bonding, and the molecule become a highly polarized because of the exchange of π-electron cloud from donor to acceptor.

**Table 8:** Second order perturbation theory analysis of Fock matrix in NBO basis of compound **3**.

Donor NBO (*i*)	Acceptor NBO (*j*)	*E*^*(2)*a^ (kJ/mol)	*E*(*j*) − *E*(*i*)^b^ (a.u.)	*F(i,j)*^c^ (a.u.)
π (C1-S3)	π* (C2-N15)	24.6856	1.08	0.071
	π* (C4-C6)	13.09592	1.2	0.055
σ (C1-C4)	π* (C1-N18)	9.95792	1.26	0.049
	π* (C4-C5)	9.99976	1.25	0.049
	π* (C4-C6)	8.5772	1.25	0.045
π (C1-N18)	σ* (C1-C4)	9.70688	1.36	0.05
	π* (C2-N19)	41.67264	0.31	0.054
	π* (C4-C5)	38.11624	0.35	0.055
π (C2-S3)	σ* (C1-C4)	17.82384	1.14	0.062
π (C2-N19)	π* (C1-N18)	58.32496	0.33	0.064
π (C4-C5)	π* (C4-C6)	13.84904	1.26	0.058
	σ* (C5-H8)	4.3932	1.16	0.031
	π* (C6-C9)	78.91024	0.29	0.067
	π* (C7-C11)	88.74264	0.27	0.068
π (C4-C6)	σ* (C1-C4)	10.20896	1.16	0.048
	σ* (C6-H10)	4.184	1.18	0.031
π (C5-C7)	π* (C4-C5)	10.96208	1.26	0.051
	σ* (C5-H8)	4.76976	1.17	0.033
	σ* (C7-H12)	4.89528	1.17	0.033
	σ* (C11 -Cl14)	19.91584	0.85	0.057
σ (C5-H8)	π* (C7-C11)	14.81136	1.08	0.055
π (C6-C9)	π* (C4-C6)	9.91608	1.25	0.049
	σ* (C6-H10)	4.85344	1.19	0.033
	σ* (C9-H13)	4.93712	1.17	0.033
	σ* (C11-Cl14)	19.83216	0.85	0.057
π (C7-C11)	π* (C5-C7)	9.28848	1.3	0.048
	σ* (C7-H12)	5.23	1.19	0.035
π (C9-C11)	π* (C6-C9)	9.07928	1.3	0.048
	σ* (C9-H13)	5.0208	1.19	0.034
σ (C11 -Cl14)	π* (C5-C7)	9.79056	1.28	0.049
	π* (C6-C9)	9.45584	1.29	0.048
LP(1) S3	π* (C1-N18)	8.5772	1.25	0.045
	π* (C2-N19)	11.04576	1.23	0.051
LP(2) S3	π* (C1-N18)	97.9056	0.26	0.07
	π* (C2-N19)	114.72528	0.25	0.075
LP(1)Cl14	π* (C7-C11)	6.6944	1.47	0.043
	π* (C9-C11)	6.6944	1.47	0.044
LP(2)Cl14	π* (C7-C11)	16.5268	0.87	0.052
	π* (C9 – C11)	16.5268	0.86	0.052
LP(3)Cl14	π* (C7-C11)	51.54688	0.32	0.061
LP(1)N15	π* (C2 – N19)	156.64896	0.29	0.099
LP(1)N18	π* (C1-S3)	69.07784	0.54	0.085
	σ* (C1-C4)	4.93712	0.85	0.029
	π* (C2 – N19)	21.17104	0.92	0.062
	σ* (C6-H10)	2.3012	0.86	0.02
LP(1)N19	π* (C1-N18)	21.58944	0.95	0.064
	π* (C2-S3)	65.35408	0.56	0.084
	π* (C2-N15)	7.7404	0.82	0.035
	σ* (N15-H16)	3.51456	0.83	0.024

^a^*E*^*(2)*^ means energy of hyper conjugative interactions (stabilization energy in kJ/mol).

^b^*Ej* − *Ei* is energy difference between donor and acceptor *i* and *j* NBO orbitals.

^c^*F(i,j)* is the Fock matrix element between *i* and *j* NBO orbitals.

### Nonlinear optical properties (NLO)

We further extended the study for the computing of polarizability (*α*) and hyperpolarizability (*β*), which were significant to envisage the nonlinear optical properties of the molecules. It is an emerging tool in telecommunications and signal processing field. In recent years, organic non-linear materials have received a considerable attention in various optoelectronic technologies. Materials of nonlinear optical properties were instigated by intramolecular charge transfer and delocalization of the electrons from the donor to acceptor electron groups in conjugated π-electron systems, which have promising materials for NLO applications^41^.

Therefore, the nonlinear optical properties of the organic π-conjugated molecule **3** were studied at the B3LYP function with the 6-31 G(d) basis set in the gas phase. The first-order hyperpolarizability (*β*_*tot*_), average linear polarizability (*α*) and total static dipole moment (*μ*) of this molecular system were predicted by using Eqs. (7, 8 and 9) and results are illustrated in Table 9.

7 $$\mu =\sqrt{({\mu }_{x}^{2}+{\mu }_{y}^{2}+{\mu }_{z}^{2})}$$

8 $$\alpha =\frac{{\alpha }_{xx}+{\alpha }_{yy}+{\alpha }_{zz}}{3}$$

and

9 $${\beta }_{tot}=\sqrt{({\beta }_{x}^{2}+{\beta }_{y}^{2}+{\beta }_{z}^{2}})$$

where

$$\begin{array}{c}{\beta }_{x}={\beta }_{xxx}+{\beta }_{xyy}+{\beta }_{xzz},\\ {\beta }_{y}={\beta }_{yyy}+{\beta }_{xxy}+{\beta }_{yzz},\\ {\beta }_{z}={\beta }_{zzz}+{\beta }_{xxz}+{\beta }_{yyz}.\end{array}$$

**Table 9:** The molecular electric dipole moments (*μ*), Polarizability (*α*) and hyperpolarizability (*β*_*tot*_) values of compound **3**.

Parameters	Values (a.u.)	Parameters	Values (a.u.)
μ_x_	−2.777	β_xxx_	−108.6
μ_y_	−2.661	β_xyy_	−40.04
μ_z_	1.053	β_xzz_	−102.2
μ	3.9875 Debye	β_yyy_	−30.59
α_xx_	244.98	β_xxy_	15.099
α_yy_	−0.241	β_yzz_	13.139
α_zz_	121.24	β_zzz_	50.252
α_xy_	−0.262	β_xxz_	−11.92
α_xz_	−0.277	β_yyz_	10.939
α_yz_	48.827	β_xyz_	5.3534
α	18.07941 × 10^−24^ esu	β_tot_	2.208 × 10^−30^ esu

For molecule **3**, the computed polarizability (*α*) and first hyperpolarizability (*β*_*tot*_) were 18.079 × 10^−24^ esu and 2.208 × 10^−30^ esu respectively. The *β*_*tot*_ of investigated compound was approximately 6 times more than for urea (0.3728 × 10^−30^ esu). Urea is a prototype NLO material and most often used reference material for comparative purpose^42^. The small energy gap (ΔE) of compound **3** indicates the occurrence of molecular charge transfer, and it can be penetrating to the stimulus of the polar environment. Thus, it led to easy electron distribution and high polarization. Small energy offers maximal NLO response^43^, hence great magnitude of the first hyperpolarizability of the title molecule might potentially serve as NLO material.

### Density of states (DOS)

**Figure 13:**
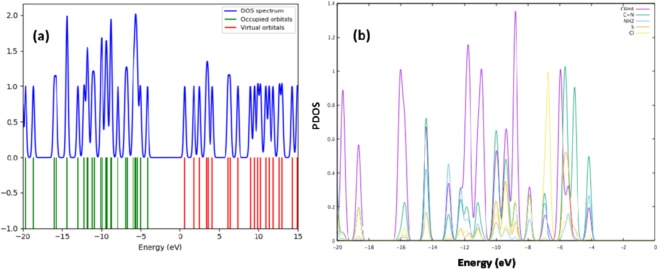
Density of sates (DOS) (**a**) and partial density of states (PDOS) (**b**) of compound **3**.

Density of states and partial density of states (PDOS) predictions were carried out by using GaussSum^43^. The TDOS plot, a population study per orbital reveals the composition of the molecular orbitals in a specific energy range very clearly (Fig. 13a). The PDOS stated on the constitutional atoms of compound **3** are illustrated in Fig. 13b and Fig. S5 (**ESI**). Five portions in the energy range from 0 to −20 eV were below the Fermi level of valence band (VB). The band (1) between −4 eV to 13.5 eV was formed by the hybridization of C 2p, N 2p, S 3p, Cl 2p and H 1 s orbitals. Likewise, the commencing energy band lying among −4 eV and the Fermi level was dominated by C 2p orbitals with partially contribution of N 2p and Cl 3p orbitals. One narrow band with a sharp peak (2) was about −14.5 eV due to the N 2p orbitals with partly aid of C 2p and S 3p orbitals. The lower parts (3–5) of the VB between −16, 18.5 and 20 eV were made up majorly of the C 2p and H 1 s orbitals as of strong hybridization of the C and H orbitals in the C_6_H_4_ group.

We detailed the experimental and theoretical characterizations of the organic compound, 1,3,4-thiadiazol-2-amine and analyzed its UV absorption as function of solvent effect. The interplay of several aspects, such as crystal structure, chemical bonding and thermodynamic properties of the compound was paramount. A good rational correlation between the suitable structural and geometrical response of experimental and theoretical parameters was observed. The compound **3** exhibited n→π* UV absorption of UV cutoff edge, and great magnitude of the first-order hyperpolarizability was observed. The obtained results suggest that compound **3** could have potential application as NLO material. Therefore, this study provides valuable insight both experimentally and theoretically, for designing new chemical entities to meet the demands of specific applications.

## Conclusions

Compound 5-(4-chlorophenyl)-2-amino-1,3,4-thiadiazole (**3**) was synthesized from 4-chlorobenzoic acid and thiosemicarbazide in POCl_3_. The structure was characterized by various spectroscopic techniques (FT-IR, NMR and HRMS) and single-crystal X-ray diffraction. XRD analysis revealed that **3** crystallizes in *Pna2*_1_ orthorhombic space group. The electronic and geometrical parameters of monomeric and dimeric forms of title molecule **3** was computed by using DFT/B3LYP/6-31+ G(d,p), B3LYP/6-31++ G(d,p) and B3LYP/6-311+G(d,p) basis sets in ground state. The observed and computed values were compared. For mono and dimeric forms energy gap (HOMO-LUMO) of compound were 3.04 eV and 2.60 eV. The low energy gap of HOMO and LUMO showed the stability of compound **3**. Moreover, the MEP shows that the positive potential sites were around the unshared electron pairs of sulfur and nitrogen atoms. Based on UV absorption the highest energy transition was found in polar solvents such as ethanol and DMSO, which resulted in increase in dipole moment (for ethanol 3.60 D and DMSO 3.65 D). In addition, the NBO analysis suggested that molecular system contains N-H…N hydrogen bonding and strong conjugative interactions and molecule become more polarized owing to the movement of π-electron cloud from donor to acceptor. The theoretical results for both basis sets were good linear agreement with the experimental X-ray crystal structure data of the title compound.

## Methods

All the chemicals (laboratory grade) and solvents were purchased from Sigma Aldrich and Merck and used without any further purification. NMR analysis was recorded on a Bruker AVANCE III 400 MHz spectrometer (399.995 MHz for ^1^H and 100.4296 MHz for ^13^C), and chemical shifts (*δ*) values were presented in parts per million (ppm). The *δ* values of DMSO-*d*_6_ representing at 2.50 ppm for ^1^H and 39.51 ppm for ^13^C NMR, were used for the structure elucidation. Functional groups of molecules were identified by infrared spectrum (IR) spectrum, which was recorded on a Perkin Elmer Spectrum 100 FT-IR Spectrometer. Further, high-resolution mass data was done by using a Bruker microTQF-Q II ESI instrument operated at ambient temperature.

A colorless plank-shaped crystal (**3**) with a dimension 0.27 × 0.17 × 0.09 mm^3^ was selected and mounted on a MITIGEN holder in paratone oil. The appropriate crystal was recorded on a ‘Bruker SMART APEX-II CCD’ single crystal diffractometer. The crystal was kept at *T* = 100 (2) K during data assortment. Data was measured using *ω* and *ϕ* scans using MoK*α* radiation. The total number of cycles and images were based on the approach calculation from the program COSMO (BRUKER) and the maximum resolution that was achieved was *Θ* = 28.415° (0.75 Å). The structure was solved with the ShelXS^44^ structure solution program using direct methods and by using Olex2^45^ as the graphical interface. The model was refined with the version 2016/6 ShelXL^46^ using Least Squares minimization. All non-hydrogen atoms were refined anisotropically. Hydrogen atom positions were intended geometrically and refined using the riding model.

The molecular structure in ground state of the title compound was fully optimized using density functional theory (DFT). B3LYP functional^47^ in combination with 6-31+ G(d,p), 6-31++ G(d,p), and 6-311+ G(d,p) basis set was employed. The optimization was performed both in gas and solvent phases. The molecular electrostatic potential map (MEP) was also predicted by using the Merz−Kollman procedure^48^ at the 6-31+ G(d,p) level and with iso-surface of the electron density (0.004 electrons per Å^3^). The ^1^H and ^13^C chemical shifts, derived on a *δ*-scale in relation to the TMS, (reference) were calculated *via* a method developed by Gregory *et. al*.^49^ The Gauge-Independent Atomic Orbital (GIAO) at B3LYP/6-31+ G(d,p) was applied for estimating the ^1^H and ^13^C NMR of the DMSO solvent. SCRF-TD-DFT/B3LYP/6-31++ G(d,p) was used for calculating the UV-electronic absorption spectrum. Frontier molecular orbital (FMO), associated to the highest molecular orbital (HOMO) and the lowest molecular orbital (LUMO) calculations were done to determine the reactivity nature^50,51^ of the compound **3**. B3LYP/sto-3g basis set and GaussSum^43^ was used for calculating the density of states spectra. The Gaussian03 program package and Gauss-View molecular visualization program was used for all the calculations^52^.

### Procedure for the synthesis of 5-(4-chlorophenyl)-2-amino-1,3,4-thiadiazole (3)

A mixture of 4-chlorobenzoic acid **1** (0.10 mmol) and thiosemicarbazide **1** (0.10 mmol) in 30 mL of phosphorous oxychloride was added dropwise with continuously stirring and refluxed gently for 1 h. The reaction mixture was permitted to cool and followed by carefully addition of water (50 mL). Again, the reaction mixture was refluxed for another 3 h. The movement of the reaction was monitored by TLC. After the completion of the reaction, the reaction mixture was basified with aqueous 30% NaOH (pH = 8). The separated solid was filtered and washed with cold water and the obtained white solid was recrystallized with hot ethanol.

White solid; Yield: 82%; mp: 148–150 ^°^C; IR cm^−1^: 33082 (=C-H), 1629 (C=N), 1597 (C=C), 1261 (=C-N), 735 (C-S-C), 706 (C-Cl); ^1^H NMR (400 MHz, DMSO-*d*_6_) *δ* 7.77 (d, J = 8.6 Hz, 2 H), 7.52 (d, J = 8.6 Hz, 2 H), 7.46 (s, 2 H); ^13^C NMR (101 MHz, DMSO-*d*_6_) *δ* 168.81, 155.12, 133.94, 129.85, 129.13, 127.89; HRMS of [C_8_H_6_N_3_SCl + H]^+^ (*m/z*): 212.0585; Calcd: 212.0591.

## Supplementary information

Supplementary Information
