# Supplementary material for: A comparison between observed and DFT calculations on structure of 5-(4-chlorophenyl)-2-amino-1,3,4-thiadiazole
Source: Sci Rep. 2019 Dec 17;9:19280. doi: 10.1038/s41598-019-55793-5 (PMC6917775; doi:10.1038/s41598-019-55793-5)
Supplement: Supplementary file 1 — Supplementary Information [file 41598_2019_55793_MOESM1_ESM.docx]

**A comparison between observed and DFT calculations on structure of 5-(4-chlorophenyl)-2-amino-1,3,4-thiadiazole**

Nagaraju Kerru, Lalitha Gummidi, Sandeep V H S Bhaskaruni, Surya Narayana Maddila, Parvesh Singh & Sreekantha B. Jonnalagadda*

**School of Chemistry & Physics, University of KwaZulu-Natal, Westville Campus, Chiltern Hills, P/Bag X54001, Durban-4000, South Africa.*

***** *Correspondence and requests for materials should be addressed to*

Prof. Sreekantha B. Jonnalagadda

School of Chemistry & Physics,

University of KwaZulu-Natal,

Durban 4000, South Africa.

Tel.: +27 31 2607325,

Fax: +27 31 2603091

E-mail: [**jonnalagaddas@ukzn.ac.za**](mailto:jonnalagaddas@ukzn.ac.za)

| **Contents** | **Pages** |
| --- | --- |
| **Materials, methods and instruments** | **2-3** |
| **1HN 13C NMR data and Atom charge transfer data (TableS1 to TableS4)** | **4-5** |
| **FMO orbitals, Mulliken, APT and PDOS plots (FigureS1 to FigureS5)** | **6-7** |
| **Experimental spectral information (FigureS6 to FigureS9)** | **8-9** |
| **Theoretical spectral information (FigureS10 to FigureS12)** | **10-11** |
| **X-ray crystal data** | **12-17** |
| **DFT optimized data** | **18-21** |

**Materials and methods**

All chemicals (laboratory grade) and solvents were purchased from Sigma Aldrich and Merck, and used without any further purification. In this study several characterization studies were conducted. NMR analysis was recorded on a Bruker AVANCE III 400 MHz spectrometer (399.995 MHz for ^1^H and 100.4296 MHz for ^13^C), and chemical shifts (δ) values were presented in parts per million (ppm). The δ values of DMSO-*d_6_* represented to at 2.50 ppm for ^1^H and 39.51 ppm for ^13^C NMR was used to analyze the structure elucidation. An infrared spectrum (IR) was recorded on a Perkin Elmer Spectrum 100 FT-IR spectrometer with universal ATR sampling accessory was used to analyze the functional groups of compound. Further, high-resolution mass data were obtained by using a Bruker microTQF-Q II ESI instrument operated at ambient temperatures.

A colourless plank-shaped crystal (**3**) with dimensions 0.27×0.17×0.09 mm^3^ was selected and mounted on a MITIGEN holder in paratone oil. A suitable crystal was recorded on a 'Bruker SMART APEX-II CCD' single crystal diffractometer. The crystal was kept at *T* = 100 (2) K during data collection. Data were measured using ω and φ scans using MoKα radiation. The total number of runs and images was based on the strategy calculation from the program COSMO (BRUKER) and the maximum resolution that was achieved was Θ = 28.415° (0.75 Å). The structure was solved with the ShelXS^46^ structure solution program using direct methods and by using Olex2^47^ as the graphical interface. The model was refined with the version 2016/6 ShelXL^48^ using Least Squares minimization. All non-hydrogen atoms were refined anisotropically. Hydrogen atom positions were calculated geometrically and refined using the riding model. Hydrogen atom positions were calculated geometrically and refined using the riding model.

The molecular structure in ground state of the compound which is refined X-ray data was fully optimized using density functional theory (DFT). B3LYP functional^49,50^ in combination with 6-31+G(d,p), and 6-31++G(d,p) basis set was employed. The optimization was performed both in gas and solvent phase. The molecular electrostatic potential map (MEP) was also determined using the Merz−Kollman procedure^51^ at the 6-31+G(d,p) level of theory and with iso-surface of the electron density (0.004 electrons per Å^3^). The ^1^H and ^13^C chemical shifts, derived on a *δ*-scale in relation to the TMS, (reference) was calculated via a method developed by Wolinski et al.^52,53^ which is the Gauge-Independent Atomic Orbital (GIAO) at B3LYP/6-31+G(d,p) and was applied for estimating the ^1^H and ^13^C NMR (DMSO solvent). SCRF-TD-DFT/B3LYP/6-31++G(d,p) was used for calculating the electronic absorption spectrum. Frontier molecular orbital (FMO) associated to the highest molecular orbital (HOMO) and the lowest molecular orbital (LUMO) calculations were done to determine the reactivity nature^54,55^ of the compound **3**. B3LYP/sto-3g basis set and GaussSum^45^ was used for calculating the density of states spectra. We used Gaussian03 program package and Gauss-View molecular visualization program for all the calculations^56^.

*Procedure for the synthesis of 5-(4-chlorophenyl)-1,3,4-thiadiazol-2-amine (****3****)*

A mixture of 4-chlorobenzoic acid**1** (0.10 mmol)and thiosemicarbazide**1** (0.10 mmol) in 30 mL of phosphorousoxychloride was added drop wisewith continuously stirring, and were refluxed gently for 1 h*.* The reaction mixture was permitted to cool, and followed by carefully addition of water (50 mL). Then after the reaction mixture was refluxed for another3 h. The movement of the reaction was monitored by TLC. Afterward, the completion of the reaction and basified with aqueous 30% NaOH (pH = 8) was added to the reaction mixture. Then the separated solid was filtered and washed with cold water. The obtained white sold was recrystallized with hot ethanol.

IR cm^-1^: 33082 (=C-H), 1629 (C=N), 1597 (C=C), 1261 (=C-N), 735 (C-S-C), 706 (C-Cl).

^1^H NMR (400 MHz, DMSO-*d_6_*) δ7.77 (d, J = 8.6 Hz, 2H), 7.52 (d, J = 8.6 Hz, 2H), 7.46 (s, 2H). ^13^C NMR (101 MHz, DMSO-*d_6_*) δ 168.81, 155.12, 133.94, 129.85, 129.13, 127.89.

HRMS of [C_8_H_6_N_3_SCl + H]^+^ (m/z): 212.0585; Calcd: 212.0591.

**Table S1**. Experimental and theoretical (B3LYP/6-31+G(d,p)) ^1^H and ^13^C chemical shift values (with respect to TMS, all values in ppm) for the compound **3**.

| **Atoms** | **Experimental Chemical shift (ppm)** | **Calculated**  **Chemical shift (ppm)** |
| --- | --- | --- |
| H1A | 7.46 | 3.12 |
| H1B | 7.46 | 3.13 |
| H4 | 7.77 | 9.09 |
| H5 | 7.52 | 8.10 |
| H7 | 7.52 | 7.96 |
| H8 | 7.77 | 8.39 |
| C4 | 127.89 | 111.59 |
| C8 | 127.89 | 114.58 |
| C5 | 129.13 | 117.32 |
| C7 | 129.13 | 116.78 |
| C3 | 129.85 | 119.67 |
| C6 | 133.94 | 132.11 |
| C1 | 155.12 | 170.46 |
| C2 | 168.81 | 166.81 |

**Table S2**. Mulliken and APT charge distribution of compound **3** monomeric form.

| **S.No** | **Atom numbers** | **Mulliken** | **APT** |
| --- | --- | --- | --- |
| 1 | 1C | -0.16771 | 0.377958 |
| 2 | 2C | -0.06554 | 0.901426 |
| 3 | 3S | 0.37607 | -0.19939 |
| 4 | 4C | -0.03302 | 0.043351 |
| 5 | 5C | -1.26375 | -0.02222 |
| 6 | 6C | 0.579398 | -0.04255 |
| 7 | 7C | 0.070491 | -0.15278 |
| 8 | 8H | 0.125702 | 0.043251 |
| 9 | 9C | -0.22282 | -0.11986 |
| 10 | 10H | 0.15885 | 0.080506 |
| 11 | 11C | -0.08226 | 0.478462 |
| 12 | 12H | 0.146204 | 0.058091 |
| 13 | 13H | 0.148482 | 0.056832 |
| 14 | 14Cl | 0.263208 | -0.40677 |
| 15 | 15N | -0.47195 | -0.84229 |
| 16 | 16H | 0.322236 | 0.237568 |
| 17 | 17H | 0.298055 | 0.229196 |
| 18 | 18N | 0.009038 | -0.09646 |
| 19 | 19N | -0.19068 | -0.62432 |

**Table S3**. Mulliken and APT charge distribution of compound **3** dimeric form.

| **S.No** | **Atom numbers** | **Mulliken** | **APT** |
| --- | --- | --- | --- |
| 1 | 1C | 0.163254 | -0.15757 |
| 2 | 2C | -1.23085 | -0.02162 |
| 3 | 3C | -0.00435 | 0.041189 |
| 4 | 4C | 0.55192 | -0.04132 |
| 5 | 5C | -0.26724 | -0.11949 |
| 6 | 6C | -0.06642 | 0.486333 |
| 7 | 7H | 0.146437 | 0.058144 |
| 8 | 8H | 0.125212 | 0.041799 |
| 9 | 9H | 0.158262 | 0.08136 |
| 10 | 10H | 0.148142 | 0.054847 |
| 11 | 11C | -0.1671 | 0.397095 |
| 12 | 12S | 0.418485 | -0.1965 |
| 13 | 13C | -0.31288 | 0.963545 |
| 14 | 14Cl | 0.262913 | -0.41435 |
| 15 | 15N | 0.052444 | -0.10083 |
| 16 | 16N | -0.22324 | -0.65945 |
| 17 | 17N | -0.40789 | -0.94361 |
| 18 | 18H | 0.306393 | 0.307476 |
| 19 | 19H | 0.320524 | 0.22737 |
| 20 | 20C | 0.77163 | -0.06112 |
| 21 | 21C | -0.25536 | -0.10911 |
| 22 | 22C | 0.076055 | 0.464067 |
| 23 | 23C | -0.26829 | -0.14423 |
| 24 | 24C | -1.19788 | -0.03934 |
| 25 | 25C | -0.18534 | 0.084864 |
| 26 | 26H | 0.159992 | 0.081164 |
| 27 | 27H | 0.148658 | 0.058847 |
| 28 | 28H | 0.163801 | 0.093421 |
| 29 | 29H | 0.129746 | 0.047747 |
| 30 | 30C | -0.08724 | 0.336213 |
| 31 | 31C | -0.03305 | 0.904012 |
| 32 | 32N | -0.4715 | -0.84504 |
| 33 | 33H | 0.322662 | 0.239741 |
| 34 | 34H | 0.299004 | 0.227271 |
| 35 | 35N | 0.012747 | -0.07171 |
| 36 | 36N | -0.19132 | -0.62957 |
| 37 | 37S | 0.373655 | -0.19533 |
| 38 | 38Cl | 0.258004 | -0.44632 |

**Table S4**. Percentage contribution of natural population analysis and natural Lewis structure of the titled compound at 6-31+G(d,p) level of theory.

|  | **Total electron density** | |
| --- | --- | --- |
| Core | 41.98876 | (99.973% of 42) |
| Valence Lewis | 63.53246 | (96.261% of 66) |
| Total Lewis | 105.52122 | (97.705% of 108) |
| Valence non-Lewis | 2.29697 | (2.127% of 108) |
| Rydberg non-Lewis | 0.18181 | (0.168% of 108) |
| Total non-Lewis | 2.47878 | (2.295% of 108) |
| Natural Minimal Basis | 107.67529 | (99.6993% of 108) |
| Natural Rydberg Basis | 0.32471 | (0.3007% of 108) |


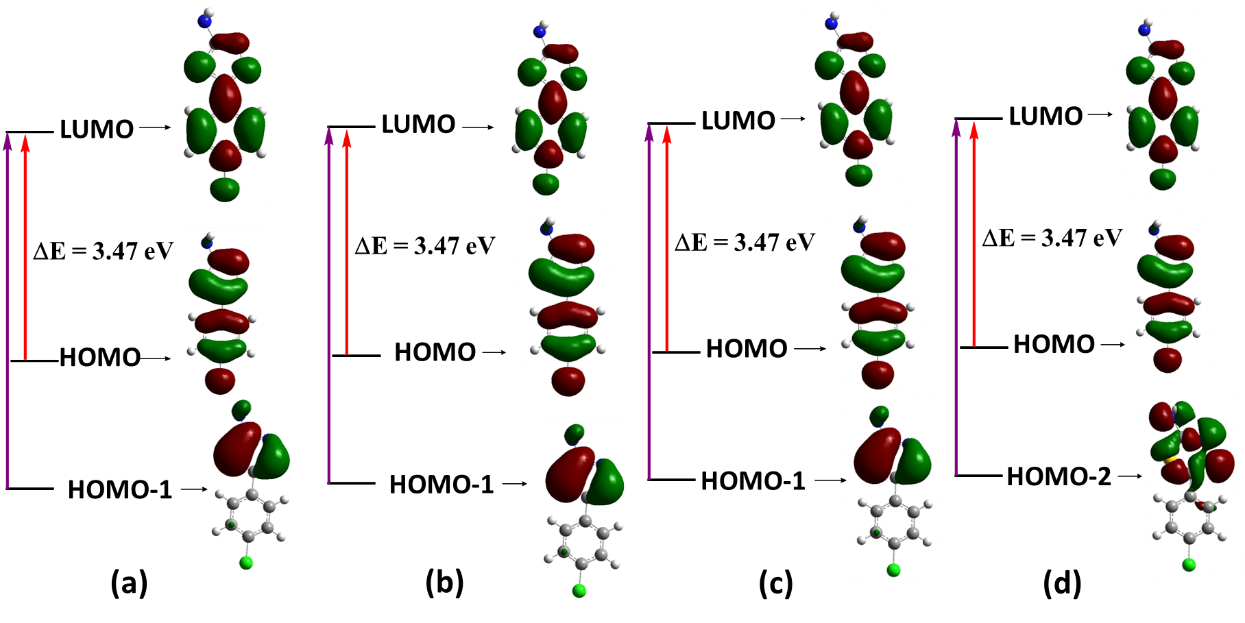


**Figure S1.** Frontier molecular orbitals contributing in electronic absorption along with band gap (ΔE) in the gas phase (**a**) and solvent phase (**b)** hexane, (**c**) acetonitrile and (**b**) ethanol for the titled compound calculated at TD-DFT/B3LYP/6-31++G(d,p) level of theory.


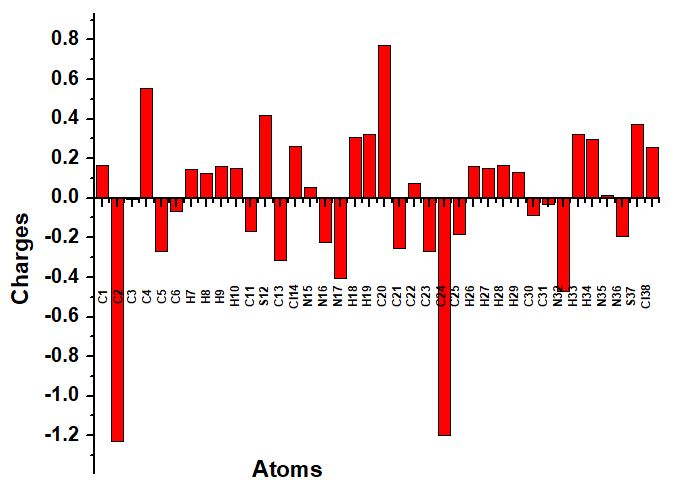


**Figure S2.** Bar diagram representing the charge distribution of compound **3** dimeric form.


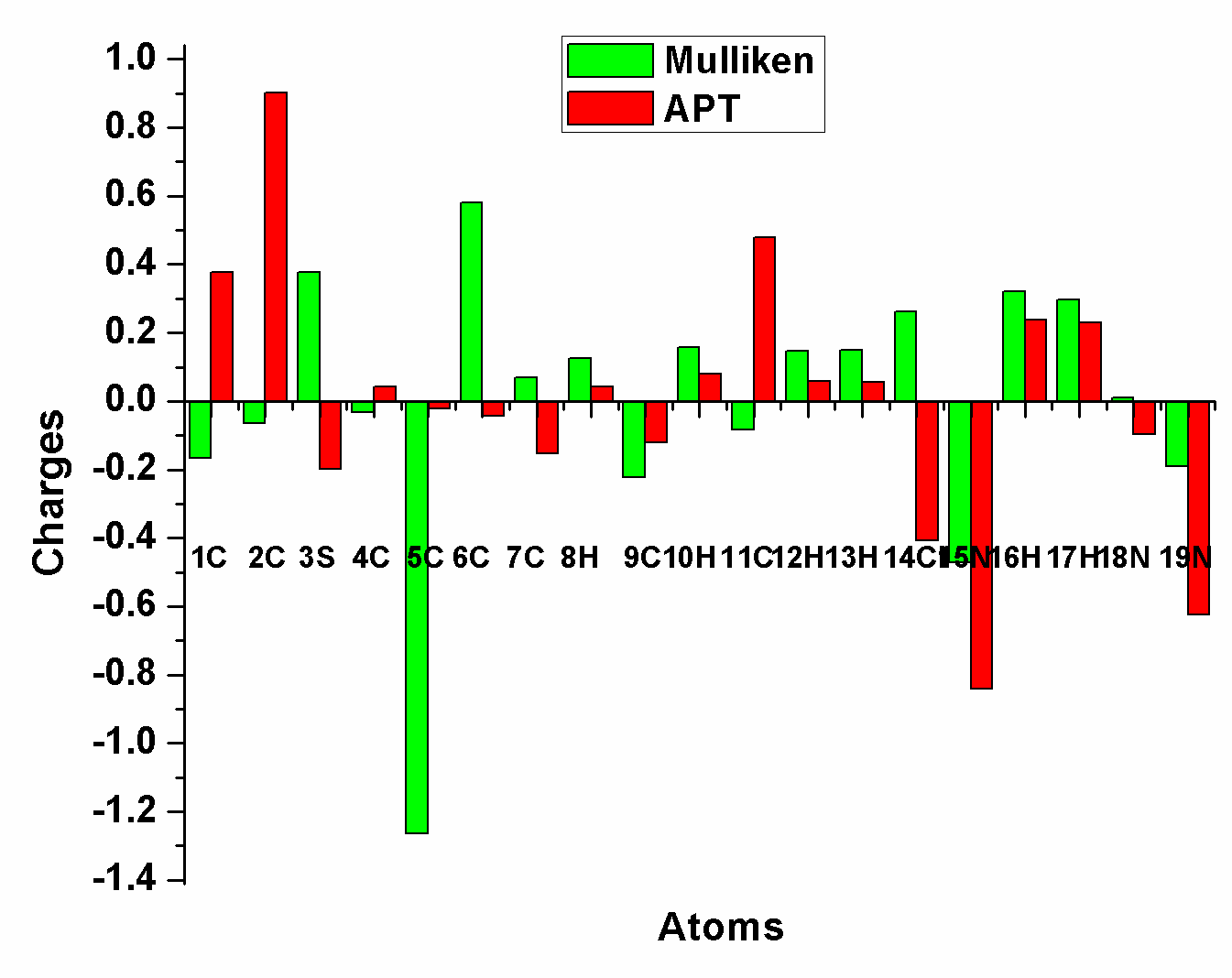


**Figure S3.** Bar diagram representing the Mulliken and APT charge distribution of compound **3** monomeric form.


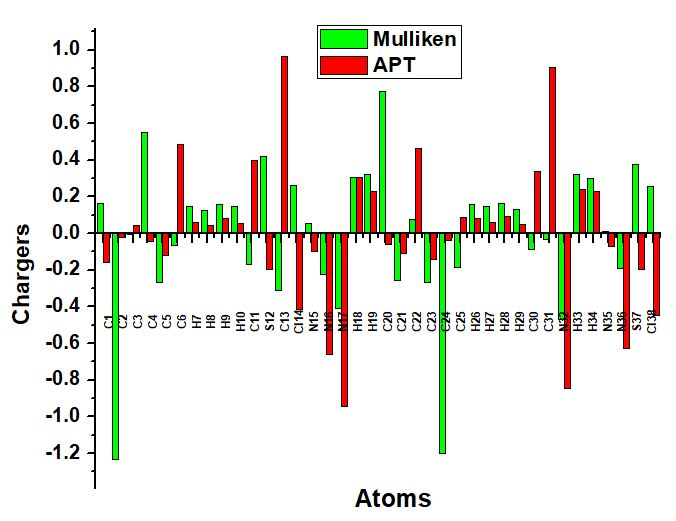


**Figure S4.** Bar diagram representing the Mulliken and APT charge distribution of compound **3** dimeric form.


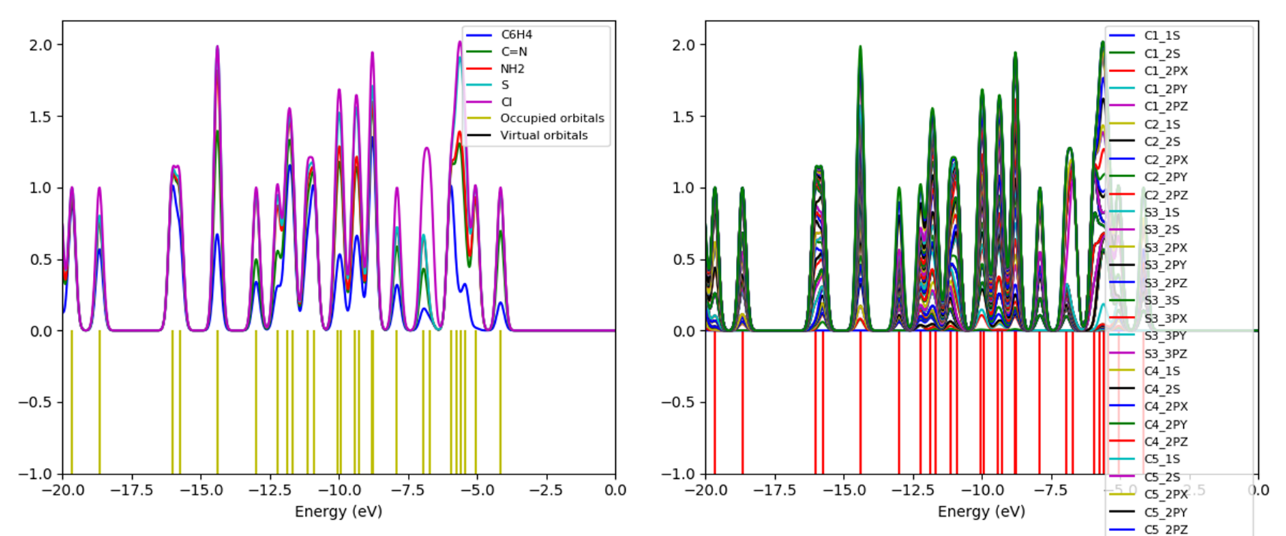


**Figure S5**. Partial density of states (PDOS) of compound **3**

**Spectras**

**Figure S6.** ^1^H-NMR spectra of compound **3**(Experimental)

**Figure S7.**^13^C-NMR spectra of compound **3** (Experimental)

**Figure S8.** IR spectra of compound **3** (Experimental)


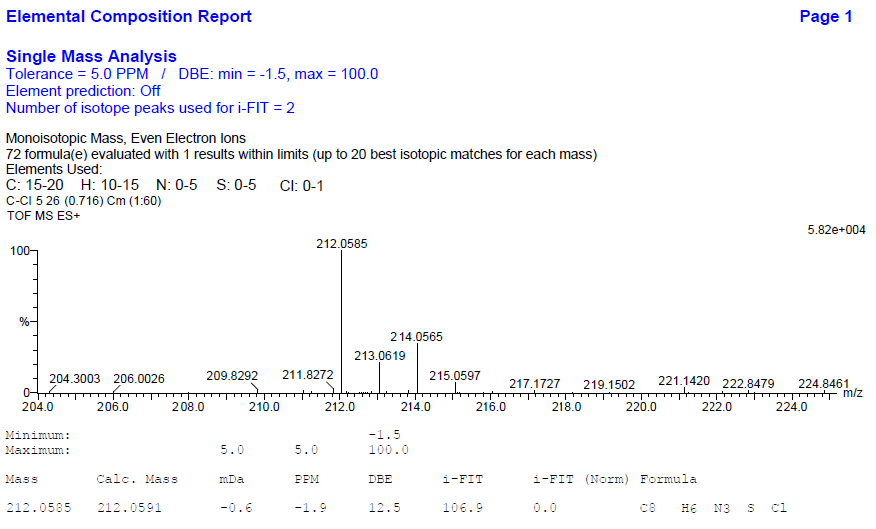


**Figure S9.** HRMS spectra of compound **3** (Experimental)


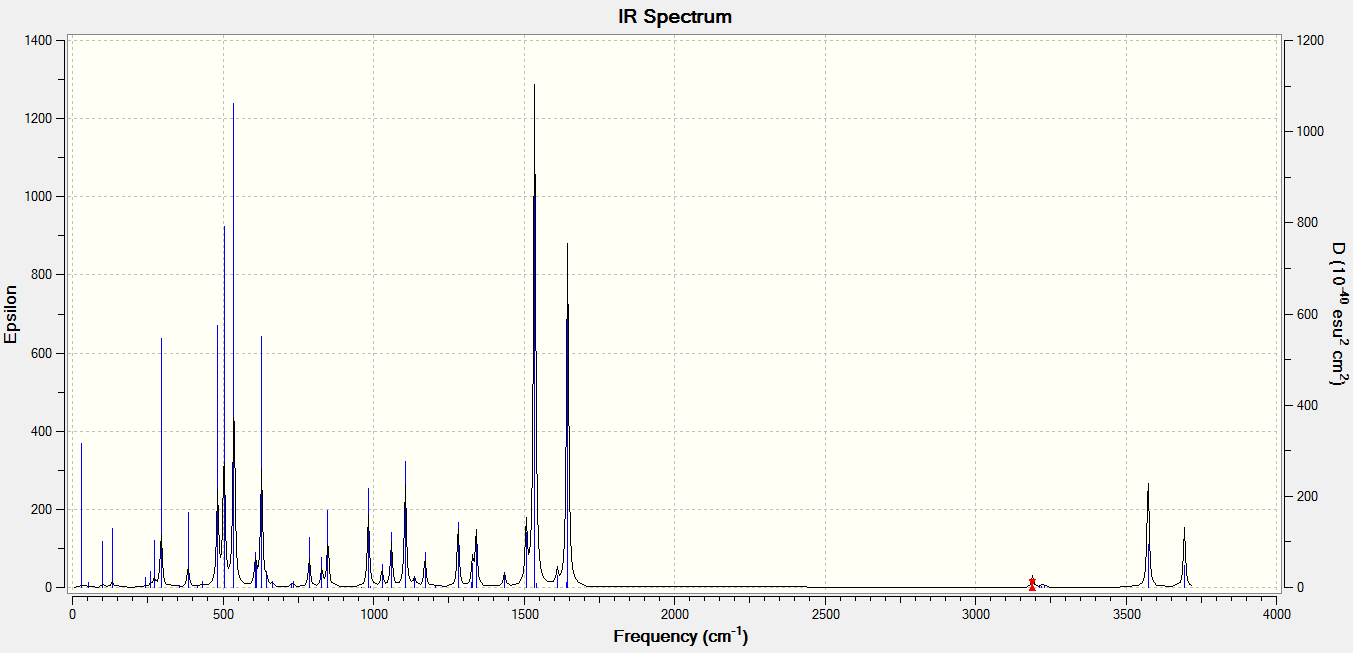


**Figure S10.** IR spectra of compound **3** (Theoretical)


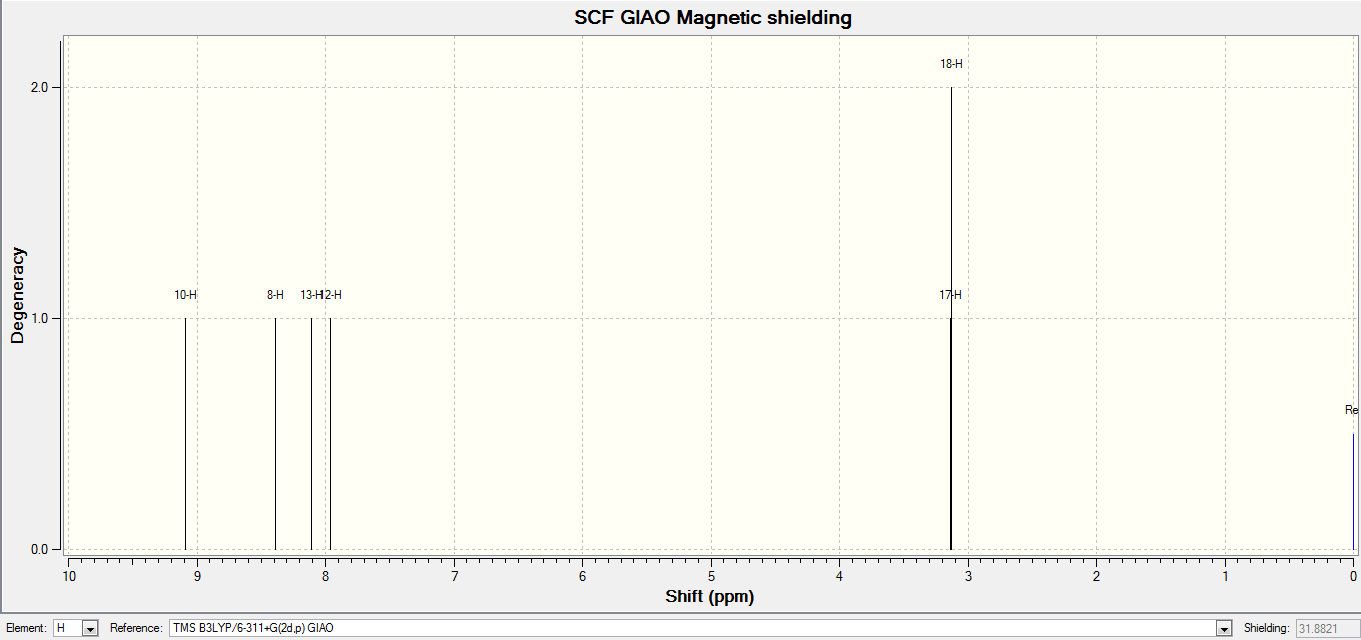


**Figure S11.** ^1^H-NMR spectra of compound **3** (Theoretical)


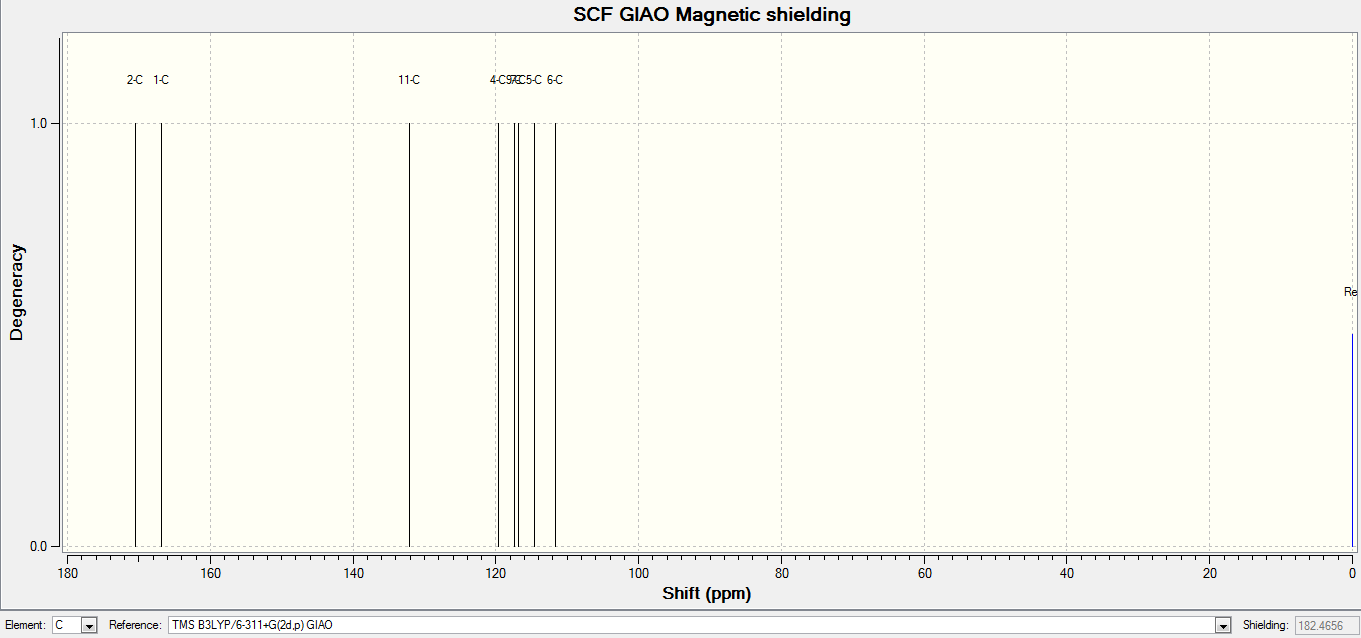


**Figure S12.** ^13^C-NMR spectra of compound **3** (Theoretical)

**X-ray crystal data.** C_8_H_6_ClN_3_S, *M_r_* = 211.67, orthorhombic, *Pna*2_1_ (No. 33), a = 11.2027(2) Å, b = 7.6705(2) Å, c = 21.2166(6) Å, *α* = *β* = *γ* = 90^°^, *V* = 1823.15(8) Å^3^, *T* = 100(2) K, *Z* = 8, *Z'* = 2, *μ*(MoK*_α_*) = 0.599, 7100 reflections measured, 3123 unique (*R_int_* = 0.0279) which were used in all calculations. The final *wR_2_* was 0.1037 (all data) and *R_1_* was 0.0401 (I > 2(I)).

**Structure Quality Indicators**

| **Reflections:** | 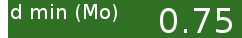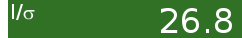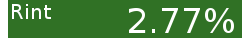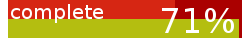 |
| --- | --- |
| **Refinement:** | 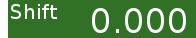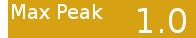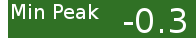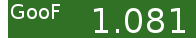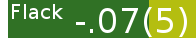 |

A colourless plank-shaped crystal with dimensions 0.27×0.17×0.09 mm^3^ was mounted on a MITIGEN holder in paratone oil. Data were collected using an Bruker SMART APEX2 area detector diffractometer equipped with an Oxford Cryosystems low-temperature device operating at *T* = 100(2) K.

Data were measured using *ω* and *φ* scans using MoK*_α_* radiation. The total number of runs and images was based on the strategy calculation from the program **COSMO** (BRUKER, V1.61, 2009) The maximum resolution that was achieved was *Θ* = 28.415^°^ (0.75 Å).

The diffraction pattern was indexed The total number of runs and images was based on the strategy calculation from the program **COSMO** (BRUKER, V1.61, 2009) and the unit cell was refined using **SAINT** (Bruker) on 3809 reflections, 54% of the observed reflections.

Data reduction, scaling and absorption corrections were performed using **SAINT** (Bruker). The final completeness is 98.60 % out to 28.415^°^ in *Θ*. A multi-scan absorption correction was performed using **<b>SADABS**</b> (Bruker). The absorption coefficient *μ* of this material is 0.599 mm^-1^ at this wavelength (*λ* = 0.711Å) and the minimum and maximum transmissions are 0.651 and 0.746.

The structure was solved and the space group *Pna*2_1_ (# 33) determined by the **ShelXS-2013** (Sheldrick, 2008) structure solution program using ? methods and refined by Least Squares using version 2016/6 of **ShelXL** (Sheldrick, 2015). All non-hydrogen atoms were refined anisotropically. Hydrogen atom positions were calculated geometrically and refined using the riding model. Hydrogen atom positions were calculated geometrically and refined using the riding model.

**Table 1**: Fractional Atomic Coordinates (×10^4^) and Equivalent Isotropic Displacement Parameters (Å^2^×10^3^) for **3**. *U_eq_* is defined as 1/3 of the trace of the orthogonalised*U_ij_*.

| **Atom** | **x** | **y** | **z** | ***U_eq_*** |
| --- | --- | --- | --- | --- |
| S1 | 2682.1(7) | 9005.0(10) | 9182.7(5) | 18.7(2) |
| Cl1 | -159.8(8) | 16305.2(12) | 10517.6(5) | 29.0(2) |
| S3 | 30.8(7) | 11050.6(11) | 7511.6(5) | 19.8(2) |
| Cl2 | 3168.6(9) | 4050.8(13) | 6099.6(6) | 33.7(3) |
| N1 | 2905(3) | 5989(4) | 8540.9(18) | 22.6(7) |
| N2 | 996(2) | 7178(4) | 8690.1(16) | 18.8(6) |
| N4 | -233(3) | 14060(4) | 8161(2) | 27.7(8) |
| N5 | 1690(2) | 12933(4) | 8000.3(17) | 19.6(6) |
| N6 | 2252(2) | 11535(4) | 7722.1(16) | 18.7(6) |
| C1 | 2161(3) | 7204(4) | 8769.2(19) | 18.5(7) |
| C2 | 1197(3) | 9669(4) | 9231.3(19) | 16.6(6) |
| C3 | 856(3) | 11284(4) | 9552.2(19) | 17.5(7) |
| C4 | -244(3) | 11432(5) | 9859.9(19) | 20.5(7) |
| C5 | -567(3) | 12974(5) | 10154(2) | 24.5(8) |
| C6 | 219(3) | 14375(5) | 10151(2) | 23.2(8) |
| N3 | 453(3) | 8602(4) | 8966.8(16) | 19.4(6) |
| C7 | 1325(3) | 14244(5) | 9855(2) | 21.6(7) |
| C8 | 1626(3) | 12717(5) | 9558.3(19) | 20.2(7) |
| C9 | 515(3) | 12858(4) | 7936.9(19) | 18.6(7) |
| C10 | 1526(3) | 10442(5) | 7450.6(19) | 17.9(7) |
| C11 | 1914(3) | 8866(4) | 7113.9(19) | 19.0(7) |
| C12 | 1218(3) | 7361(5) | 7124(2) | 22.1(7) |
| C13 | 1600(3) | 5865(5) | 6816(2) | 25.5(8) |
| C14 | 2676(3) | 5904(5) | 6494(2) | 24.2(8) |
| C16 | 2991(3) | 8876(5) | 6782(2) | 23.4(8) |
| C15 | 3366(3) | 7384(6) | 6474(2) | 27.0(8) |

**Table 2**: Anisotropic Displacement Parameters (×10^4^) **3**. The anisotropic displacement factor exponent takes the form: *-2π^2^[h^2^a*^2^ × U_11_+ ... +2hka* × b* × U_12_]*

| **Atom** | ***U_11_*** | ***U_22_*** | ***U_33_*** | ***U_23_*** | ***U_13_*** | ***U_12_*** |
| --- | --- | --- | --- | --- | --- | --- |
| S1 | 9.8(3) | 21.0(4) | 25.3(5) | -0.7(4) | -2.2(3) | -1.8(3) |
| Cl1 | 29.2(4) | 25.3(4) | 32.3(6) | -8.7(4) | -2.8(4) | 0.4(3) |
| S3 | 10.3(3) | 24.0(4) | 25.0(5) | -4.4(4) | -1.4(3) | -1.1(3) |
| Cl2 | 36.1(5) | 35.8(5) | 29.2(6) | -10.9(4) | -3.9(5) | 15.0(4) |
| N1 | 9.6(12) | 23.4(15) | 35(2) | -7.7(13) | -3.5(13) | 0.7(10) |
| N2 | 11.3(11) | 19.6(14) | 25.4(18) | -1.5(12) | -2.8(12) | 0.8(10) |
| N4 | 10.4(13) | 27.2(17) | 45(2) | -11.2(15) | -1.8(14) | 1.2(11) |
| N5 | 11.2(11) | 22.9(14) | 24.8(18) | -3.0(13) | -3.3(12) | 1.5(10) |
| N6 | 11.3(12) | 23.6(14) | 21.2(16) | 0.4(12) | -0.8(12) | 0.7(10) |
| C1 | 12.1(13) | 21.1(17) | 22(2) | -0.6(14) | -2.5(14) | -2.2(11) |
| C2 | 10.8(13) | 18.7(15) | 20.3(18) | 3.2(14) | -1.9(13) | -1.0(10) |
| C3 | 13.1(14) | 19.4(16) | 20.1(19) | 0.1(13) | -2.3(13) | 1.4(11) |
| C4 | 13.2(14) | 25.0(17) | 23(2) | -1.5(15) | 0.2(14) | -2.7(12) |
| C5 | 17.1(15) | 29.9(19) | 26(2) | -4.8(15) | -0.8(15) | -2.3(13) |
| C6 | 23.3(16) | 23.3(17) | 23(2) | -2.4(15) | -5.6(15) | 4.5(14) |
| N3 | 12.0(12) | 23.9(14) | 22.3(17) | 1.2(12) | -1.3(12) | -0.4(10) |
| C7 | 21.1(16) | 21.9(17) | 22(2) | 2.3(14) | -3.7(14) | -4.7(13) |
| C8 | 15.7(14) | 23.0(17) | 22(2) | 1.9(14) | -2.4(14) | -1.4(12) |
| C9 | 13.0(13) | 22.1(16) | 20.8(19) | -2.8(14) | -0.1(13) | -1.7(11) |
| C10 | 11.3(13) | 22.9(16) | 19.4(19) | 1.6(14) | 1.5(13) | 0.2(11) |
| C11 | 15.4(15) | 25.1(17) | 16.5(19) | 0.3(14) | -1.7(14) | 3.0(12) |
| C12 | 22.5(16) | 27.0(17) | 16.9(19) | -2.0(15) | 2.9(14) | -2.5(13) |
| C13 | 28.4(18) | 26.6(19) | 21(2) | -0.8(15) | 1.4(17) | -1.7(14) |
| C14 | 25.3(18) | 31(2) | 16.4(19) | -2.8(15) | -2.9(16) | 9.6(14) |
| C16 | 16.7(15) | 24.5(17) | 29(2) | 1.5(15) | 2.8(16) | -1.2(12) |
| C15 | 18.3(14) | 42(2) | 21(2) | -2.9(17) | 3.1(16) | 3.8(15) |

**Table 3**: Bond Lengths in Å for **3**.

| **Atom** | **Atom** | **Length/Å** |
| --- | --- | --- |
| S1 | C1 | 1.738(4) |
| S1 | C2 | 1.743(3) |
| Cl1 | C6 | 1.725(4) |
| S3 | C9 | 1.741(4) |
| S3 | C10 | 1.744(3) |
| Cl2 | C14 | 1.739(4) |
| N1 | C1 | 1.340(4) |
| N2 | C1 | 1.316(4) |
| N2 | N3 | 1.381(4) |
| N4 | C9 | 1.333(5) |
| N5 | N6 | 1.377(4) |
| N5 | C9 | 1.324(4) |
| N6 | C10 | 1.303(4) |
| C2 | C3 | 1.464(5) |
| C2 | N3 | 1.296(4) |
| C3 | C4 | 1.399(5) |
| C3 | C8 | 1.398(5) |
| C4 | C5 | 1.386(5) |
| C5 | C6 | 1.389(5) |
| C6 | C7 | 1.393(5) |
| C7 | C8 | 1.371(5) |
| C10 | C11 | 1.469(5) |
| C11 | C12 | 1.393(5) |
| C11 | C16 | 1.397(5) |
| C12 | C13 | 1.388(5) |
| C13 | C14 | 1.386(6) |
| C14 | C15 | 1.375(6) |
| C16 | C15 | 1.384(6) |

**Table 4**: Bond Angles in ^°^ for **3**.

| **Atom** | **Atom** | **Atom** | **Angle/^°^** |
| --- | --- | --- | --- |
| C1 | S1 | C2 | 86.66(16) |
| C9 | S3 | C10 | 87.27(16) |
| C1 | N2 | N3 | 111.8(3) |
| C9 | N5 | N6 | 112.2(3) |
| C10 | N6 | N5 | 113.9(3) |
| N1 | C1 | S1 | 121.8(2) |
| N2 | C1 | S1 | 114.2(3) |
| N2 | C1 | N1 | 124.0(3) |
| C3 | C2 | S1 | 121.6(2) |
| N3 | C2 | S1 | 113.8(3) |
| N3 | C2 | C3 | 124.6(3) |
| C4 | C3 | C2 | 121.0(3) |
| C8 | C3 | C2 | 120.6(3) |
| C8 | C3 | C4 | 118.4(3) |
| C5 | C4 | C3 | 120.6(3) |
| C4 | C5 | C6 | 119.5(3) |
| C5 | C6 | Cl1 | 120.4(3) |
| C5 | C6 | C7 | 120.7(4) |
| C7 | C6 | Cl1 | 118.9(3) |
| C2 | N3 | N2 | 113.6(3) |
| C8 | C7 | C6 | 119.2(3) |
| C7 | C8 | C3 | 121.6(3) |
| N4 | C9 | S3 | 122.6(3) |
| N5 | C9 | S3 | 113.4(3) |
| N5 | C9 | N4 | 124.0(3) |
| N6 | C10 | S3 | 113.2(3) |
| N6 | C10 | C11 | 124.1(3) |
| C11 | C10 | S3 | 122.7(3) |
| C12 | C11 | C10 | 120.6(3) |
| C12 | C11 | C16 | 119.7(3) |
| C16 | C11 | C10 | 119.7(3) |
| C13 | C12 | C11 | 120.4(4) |
| C14 | C13 | C12 | 118.8(4) |
| C13 | C14 | Cl2 | 119.7(3) |
| C15 | C14 | Cl2 | 118.8(3) |
| C15 | C14 | C13 | 121.5(4) |
| C15 | C16 | C11 | 119.8(3) |
| C14 | C15 | C16 | 119.8(4) |

**Table 4**: Torsion Angles in ^°^ for **3**.

| **Atom** | **Atom** | **Atom** | **Atom** | **Angle/^°^** |
| --- | --- | --- | --- | --- |
| S1 | C2 | C3 | C4 | -148.4(3) |
| S1 | C2 | C3 | C8 | 31.5(5) |
| S1 | C2 | N3 | N2 | -1.2(4) |
| Cl1 | C6 | C7 | C8 | -179.5(3) |
| S3 | C10 | C11 | C12 | -35.3(5) |
| S3 | C10 | C11 | C16 | 144.9(3) |
| Cl2 | C14 | C15 | C16 | 179.8(3) |
| N5 | N6 | C10 | S3 | 0.2(4) |
| N5 | N6 | C10 | C11 | 179.0(3) |
| N6 | N5 | C9 | S3 | -1.1(4) |
| N6 | N5 | C9 | N4 | -179.6(4) |
| N6 | C10 | C11 | C12 | 146.0(4) |
| N6 | C10 | C11 | C16 | -33.8(6) |
| C1 | S1 | C2 | C3 | 180.0(3) |
| C1 | S1 | C2 | N3 | 0.6(3) |
| C1 | N2 | N3 | C2 | 1.3(5) |
| C2 | S1 | C1 | N1 | 178.4(4) |
| C2 | S1 | C1 | N2 | 0.1(3) |
| C2 | C3 | C4 | C5 | -179.0(4) |
| C2 | C3 | C8 | C7 | 179.9(4) |
| C3 | C2 | N3 | N2 | 179.4(3) |
| C3 | C4 | C5 | C6 | -1.0(6) |
| C4 | C3 | C8 | C7 | -0.2(6) |
| C4 | C5 | C6 | Cl1 | -179.6(3) |
| C4 | C5 | C6 | C7 | 0.0(6) |
| C5 | C6 | C7 | C8 | 0.8(6) |
| C6 | C7 | C8 | C3 | -0.7(6) |
| N3 | N2 | C1 | S1 | -0.8(4) |
| N3 | N2 | C1 | N1 | -179.1(4) |
| N3 | C2 | C3 | C4 | 30.8(6) |
| N3 | C2 | C3 | C8 | -149.2(4) |
| C8 | C3 | C4 | C5 | 1.1(6) |
| C9 | S3 | C10 | N6 | -0.6(3) |
| C9 | S3 | C10 | C11 | -179.5(3) |
| C9 | N5 | N6 | C10 | 0.6(5) |
| C10 | S3 | C9 | N4 | 179.5(4) |
| C10 | S3 | C9 | N5 | 1.0(3) |
| C10 | C11 | C12 | C13 | -178.7(4) |
| C10 | C11 | C16 | C15 | 179.0(4) |
| C11 | C12 | C13 | C14 | -0.7(6) |
| C11 | C16 | C15 | C14 | 0.1(6) |
| C12 | C11 | C16 | C15 | -0.8(6) |
| C12 | C13 | C14 | Cl2 | -179.5(3) |
| C12 | C13 | C14 | C15 | -0.1(6) |
| C13 | C14 | C15 | C16 | 0.3(6) |
| C16 | C11 | C12 | C13 | 1.1(6) |

**Table 5**. Intermolecular hydrogen bonding geometry of the compound **3** (**Å, °**)

| **D-H…A** | **d(D-H)/Å** | **d(H…A)/Å** | **d(D…A)/Å** | **∠D-H…A/°** |
| --- | --- | --- | --- | --- |
| N(1)-H(1A)…N(5)^i^ | 0.88 | 2.08 | 2.943(4) | 166.1 |
| N(4)-H(4A)…N(2)^ii^ | 0.88 | 2.11 | 2.979(4) | 167.3 |

Symmetry codes: ^i^+X,-1+Y,+Z; ^ii^+X,1+Y,+Z

**Table 6**: Hydrogen Fractional Atomic Coordinates (×10^4^) and Equivalent Isotropic Displacement Parameters (Å^2^×10^3^) for **3**. *U_eq_* is defined as 1/3 of the trace of the orthogonalised*U_ij_*.

| **Atom** | **x** | **y** | **z** | ***U_eq_*** |
| --- | --- | --- | --- | --- |
| H1A | 2620.11 | 5105.34 | 8323.07 | 27 |
| H1B | 3677.41 | 6074.53 | 8608.83 | 27 |
| H4A | 47.44 | 14971.12 | 8364.86 | 33 |
| H4B | -1006.97 | 13942.36 | 8104.47 | 33 |
| H4 | -774.2 | 10465.52 | 9867.17 | 25 |
| H5 | -1320.21 | 13072.06 | 10356.71 | 29 |
| H7 | 1863.88 | 15200.22 | 9857.9 | 26 |
| H8 | 2376.38 | 12631.76 | 9352.21 | 24 |
| H12 | 478.53 | 7358.56 | 7343.11 | 27 |
| H13 | 1131.27 | 4832.39 | 6825.73 | 31 |
| H16 | 3464.61 | 9902.7 | 6768.88 | 28 |
| H15 | 4098.96 | 7383.22 | 6248.18 | 32 |

**DFT calculation data**

%nprocshared=24

%mem=24GB

%chk=3emono.chk

# opt freq b3lyp/6-31+g(d,p) geom=connectivity pop= (esp, nbo)

3emono

0 1

C -0.36753123 1.04216958 0.77923159

C 0.52860977 3.31449258 0.78004159

S -0.98202623 2.59733558 0.78003359

C -1.26570887 -0.20878062 0.77850843

C -2.65401653 -0.07067456 0.77858827

C -0.69132895 -1.47985699 0.77766245

C -3.46768312 -1.20344479 0.77850413

H -3.10653684 0.93155549 0.78003255

C -1.50510332 -2.61307364 0.77658227

H 0.40285621 -1.58888822 0.77741950

C -2.89308532 -2.47507840 0.77714108

H -4.56196992 -1.09466779 0.77920105

H -1.05194904 -3.61513364 0.77549997

Cl -3.92018364 -3.90429812 0.77694052

N 0.71692922 4.77237975 0.78091559

H 1.22748179 5.04204327 1.59738215

H 1.22682249 5.04310742 -0.03561083

N 1.00969477 1.04216958 0.77923159

N 1.53506977 2.37443958 0.77923159

1 3 1.0 4 1.0 18 2.0

2 3 1.0 15 1.0 19 2.0

3

4 5 1.5 6 1.5

5 7 1.5 8 1.0

6 9 1.5 10 1.0

7 11 1.5 12 1.0

8

9 11 1.5 13 1.0

10

11 14 1.0

12

13

14

15 16 1.0 17 1.0

16

17

18 19 1.0

19

%nprocshared=24

%mem=24GB

%chk=3edimer1i.chk

# opt freq b3lyp/6-31+g(d,p) pop=(esp, nbo) geom=connectivity

3edimer1i

0 1

C 0.73791240 1.70410548 -2.85725256

C 1.88820364 2.45172975 -2.60356092

C 1.81317559 3.84220525 -2.52500891

C 0.58763111 4.48575526 -2.70136271

C -0.56225835 3.73827594 -2.95546094

C -0.48721112 2.34731901 -3.03302762

H 0.79720224 0.60781566 -2.91870306

H 2.85377937 1.94428289 -2.46426635

H 0.52873030 5.58210955 -2.63944089

H -1.52827860 4.24517498 -3.09450221

C 3.08267008 4.66748145 -2.24411220

S 4.58554273 3.96869298 -2.02226885

C 5.35658919 5.43386045 -1.78752023

Cl -1.93841553 1.40445325 -3.35335543

N 3.14260131 6.03966108 -2.14270821

N 4.47572213 6.48892552 -1.87454304

N 6.79873809 5.54036631 -1.52339325

H 7.23051528 6.08241631 -2.24433032

H 6.94379372 5.98527529 -0.63964279

C 9.01444014 1.04528360 -2.83259511

C 10.09989181 1.83688983 -2.45626324

C 9.92140050 3.20044095 -2.22368862

C 8.65721415 3.77320249 -2.36858034

C 7.57216322 2.98181470 -2.74528562

C 7.75068271 1.61767432 -2.97693559

H 9.15529012 -0.02982440 -3.01548003

H 11.09593319 1.38517884 -2.34180565

H 8.51673481 4.84834712 -2.18520663

H 6.57571199 3.43292274 -2.85956904

C 6.55243996 0.74413986 -3.39256988

C 4.38843575 0.11690901 -3.95692895

N 2.95304275 0.30390327 -4.21301501

H 2.74601248 0.04359202 -5.15608262

H 2.42516402 -0.26859621 -3.58564878

N 6.68775420 -0.89614693 -3.68802774

N 5.06623774 -1.07800550 -4.05401554

S 5.25967463 1.18515956 -3.56866893

Cl 11.29042736 4.19896537 -1.74796344

1 2 1.5 6 1.5 7 1.0

2 3 1.5 8 1.0

3 4 1.5 11 1.0

4 5 1.5 9 1.0

5 6 1.5 10 1.0

6 14 1.0

7

8

9

10

11 12 1.0 15 2.0

12 13 1.0

13 16 2.0 17 1.0

14

15 16 1.0

16

17 18 1.0 19 1.0

18

19

20 21 1.5 25 1.5 26 1.0

21 22 1.5 27 1.0

22 23 1.5 38 1.0

23 24 1.5 28 1.0

24 25 1.5 29 1.0

25 30 1.0

26

27

28

29

30 35 2.0 37 1.0

31 32 1.0 36 2.0 37 1.0

32 33 1.0 34 1.0

33

34

35 36 1.0

36

37

38
